# Dust devil migration patterns reveal strong near-surface winds across Mars

**DOI:** 10.1126/sciadv.adw5170

**Published:** 2025-10-08

**Authors:** Valentin T. Bickel, Miguel Almeida, Matthew Read, Antonia Schriever, Daniela Tirsch, Ernst Hauber, Klaus Gwinner, Nicolas Thomas, Thomas Roatsch

**Affiliations:** ^1^ Center for Space and Habitability, University of Bern, Bern, Switzerland.; ^2^ Space Research and Planetary Sciences, Physics Institute, University of Bern, Bern, Switzerland.; ^3^ Centre for Earth, Planetary, Space and Astronomical Research, Open University, Milton Keynes, UK.; ^4^ Institute of Planetary Research, German Aerospace Center (DLR), Berlin, Germany.

## Abstract

Dust devil migration is a direct expression of the dynamics of the lowermost martian atmosphere. These dynamics are responsible for dust lifting and atmospheric injection, a vital part of the dust cycle that governs modern Mars’ weather and climate. Here, we use deep learning and two decades’ worth of orbital images to track the global, diurnal, and seasonal migration patterns of dust devils across Mars, providing a distributed characterization of the dynamics of near-surface winds. Across Mars, derived wind stresses systematically exceed those predicted by global circulation models and frequently surpass the threshold required to initiate particle saltation and the lifting of dust. We identify instances of fast-moving dust devils, indicating strong near-surface winds, that are colocated with large-scale dust lifting events and storms. Our observations show that strong near-surface winds are abundant on Mars and play an important role in atmospheric dust sourcing, directly informing more accurate models of Mars’ atmosphere, weather, and climate.

## Teaser

Dust devil migration reveals how the dynamics of the lowermost atmosphere drive Mars’ dust cycle, weather, and climate.

## INTRODUCTION

Dust controls numerous atmospheric and surface processes on Mars. In the atmosphere, suspended dust can lead to substantial temperature variations, can perturb atmospheric dynamics, and can lead to local, regional, and even global dust storms (*1*–*9*). On the surface, seasonal and post–storm dust settling has been tied to the occurrence and modification of surface features such as slope streaks and RSL (recurring slope lineae) (*10*–*12*). The role of dust in controlling the temperature of the atmosphere and surface makes it a central part of the weather and climate system. In addition, dust might affect Mars’ past and current habitability through the exchange and distribution of nutrients and shielding of ultraviolet radiation (*13*, *14*). Dust has a direct effect on the exploration of Mars as well, by affecting remote sensing observations, surface hardware, crew operations, and the power efficiency of solar panels; in particular, large-scale dust storms and planet-encircling dust events (PEDEs) can severely affect operations on Mars (*15*–*19*). The National Aeronautics and Space Administration’s most recent Mars Exploration Program specifically calls out the need for a better understanding of the dynamics and interactions of Mars’ dusty surface and atmosphere (Science Themes 2.C and 3.A-3.C) (*20*) in the context of sustainable, future exploration.

Most likely, martian dust (here, particles <62 μm) is the result of aeolian processes eroding exposed bedrock over geologic time frames (*21*). Past work established that wind stress exerted by (i) “vortical” processes, such as convective vortices and dust devils, and (ii) “nonvortical” processes, such as (straight-line) wind gusts, convection cell fronts, and meridional circulation, is responsible for the bulk injection of dust into the atmosphere (*3*, *8*, *14*, *22*–*29*). Dust particles are difficult to lift directly into the thin martian atmosphere due to cohesive forces, especially if they do not form dust aggregates (*30*). However, the saltation of larger grains (~100 μm, “fine-grained sand”) is easier to initiate on Mars at wind shear velocities beyond the “static threshold” (also referred to as “friction angle velocity”). In turn, saltating sand particles can directly and efficiently inject finer-sized particles (dust) into the atmosphere upon reimpact on the surface [e.g., (*31*–*36*)].

Early modeling efforts and wind tunnel experiments suggested that the static threshold of ~100- to ~200-μm-sized martian grains is located at wind shear velocities (i.e., at surface level) between ~1.5 and ~2 m/s, which can be converted to wind velocities between ~15 and ~22 m/s at an altitude of ~1.5 m above the surface, assuming an average aerodynamic surface roughness and a convectively stable atmosphere [e.g., (*37*–*39*)]. However, the atmospheric data collected by missions on Mars seemed to suggest that winds do not—or only rarely—exceed this threshold, despite frequent observations of active aeolian surface processes from orbit, a discrepancy termed the “martian sand transport puzzle” (*30*, *40*–*48*). More recent in situ measurements on Mars—most prominently by the InSight lander (Interior Exploration using Seismic Investigations, Geodesy and Heat Transport) and Perseverance rover—showed that wind velocities can be substantially higher [up to ~32 m/s at an altitude of ~1.5 m above the surface (*29*, *49*, *50*)] and that sand and dust transport can be initiated at lower wind shear velocities than anticipated earlier [at wind shear beyond ~1.2 m/s, converted from a wind velocity of ~15 m/s measured at an altitude of ~1.5 m above the surface, under the above assumptions (*29*)].

The vast majority of macroscopic sand and dust mobilization events observed by Perseverance, InSight, and other landed missions were driven by vortical winds associated with passing convective vortices and dust devils. The pressure drop that is associated with those vortical features (“suction effect”) has been shown to contribute to dust lifting and makes it challenging to use the observed wind shear velocities to constrain the static threshold directly. Direct observations of nonvortical, straight-line winds lifting dust off the surface would enable a more robust constraint of the static threshold, but have been largely missing. Very recently, Guzewich *et al.* (*8*) and Newman *et al.* (*29*) observed several dust lifting events in the direct vicinity of the Perseverance and Curiosity rovers that were driven by straight-line, nonvortical wind gusts and that affected substantially larger areas than individual dust devils. Perseverance measured a peak wind velocity of ~15 m/s (at ~1.5 m above the surface) during one of those events, although the event did not occur directly at the rover’s location. These observations suggest that nonvortical wind gusts might play an important—and potentially dominant—role in lifting dust off the martian surface, an observation that is further amplified by a more recent wind tunnel experiment that located the static threshold of 100-μm-sized grains at a much lower wind shear velocity (~0.6 m/s) than earlier wind tunnel studies and modeling efforts (*51*).

Today, it remains unclear where exactly the static threshold envelope is located and how it is shaped, what the abundance and spatiotemporal distribution of nonvortical, straight-line winds is on a global scale, and whether those nonvortical, near-surface winds—and the associated wind stresses—are strong and widespread enough to play an important role in dust lifting and, thus, the martian dust cycle overall.

Here, we use orbital observations of dust devil migration to visualize and quantify the characteristics and dynamics of nonvortical, near-surface winds in Mars’ lowermost atmosphere. We use a deep learning approach to identify more than 1000 dust devils in the European Space Agency’s (ESA’s) Trace Gas Orbiter (TGO) CaSSIS [Colour and Stereo Surface Imaging System (*52*)] and Mars Express (MEX) HRSC [High-Resolution Stereo Camera (*53*)] datasets acquired over more than 10 Mars years (MY 27 to MY 37, 2004 to 2024, with a temporal CaSSIS-HRSC overlap of currently 7 years/~4 MY). Because of the specific, non–sun-synchronous orbits of their host spacecraft and their stereo- and color-imaging capabilities, both CaSSIS and HRSC provide a unique opportunity to characterize dust devil migration—i.e., displacement velocity and azimuth—through stereo pairs and “color fringes” at second to minute timescales, as a function of local solar time (LST) and season, which is generally not possible using data from other spacecraft orbiting Mars [see (*54*)]. Here, the term “color fringe” refers to CaSSIS color-infrared image artifacts caused by dynamically moving targets such as dust devils and clouds (fig. S1). We use the unique traits of the CaSSIS and HRSC data to create a global-scale, long-term, spatiotemporal, and quantitative description of the dynamics of near-surface, nonvortical winds and correlate it with other thermophysical and atmospheric datasets as well as global-scale weather maps and Mars Climate Database [MCD (*55*)] model outputs. This work presents global-scale observational evidence that supports the critical role of nonvortical, straight-line winds in the martian dust cycle with important implications on the optimization of Global Circulation Models (GCMs) and the continued—robotic and human—exploration of planet Mars.

## RESULTS

**Fig. 1.**
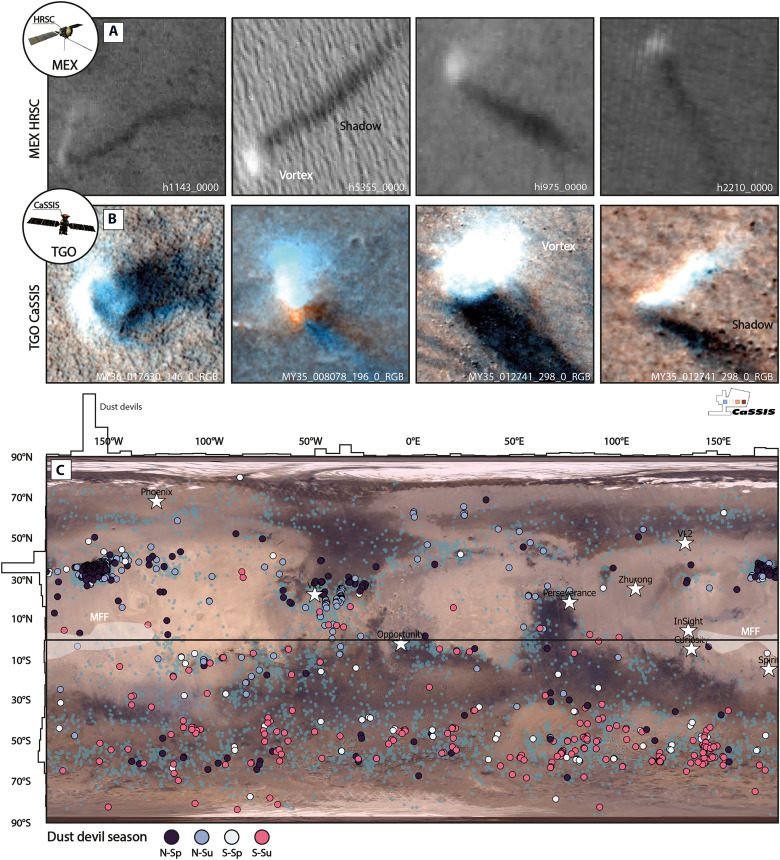
The CaSSIS and HRSC dust devil catalog. Examples of (**A**) HRSC and (**B**) CaSSIS-imaged dust devils on Mars; note color fringing in some of the CaSSIS dust devils. (**C**) Global map of dust devil distribution (*n* = 1039), symbol color indicates season of occurrence (N-Sp, northern spring; N-Su, northern summer; S-Sp, southern spring; S-Su, southern summer); histograms indicate longitudinal and latitudinal distribution. Teal dots represent CTX-derived dust devil detections recorded by an earlier survey [*n* = 12,828 (*58*)]. Locations of landed missions (white stars) and the Medusae Fossae Formation (MFF) indicated. Viking color mosaic in the background. Spacecraft badges mark data derived by different missions/instruments. Image credit: ESA/TGO/CaSSIS CC -BY-SA 3.0 (https://creativecommons.org/licenses/by-sa/3.0/deed.en) IGO, ESA/DLR/FU Berlin CC -BY-SA 3.0 (https://creativecommons.org/licenses/by-sa/3.0/deed.en) IGO; the shown images were cropped from the original CaSSIS and HRSC images.

**Fig. 2.**
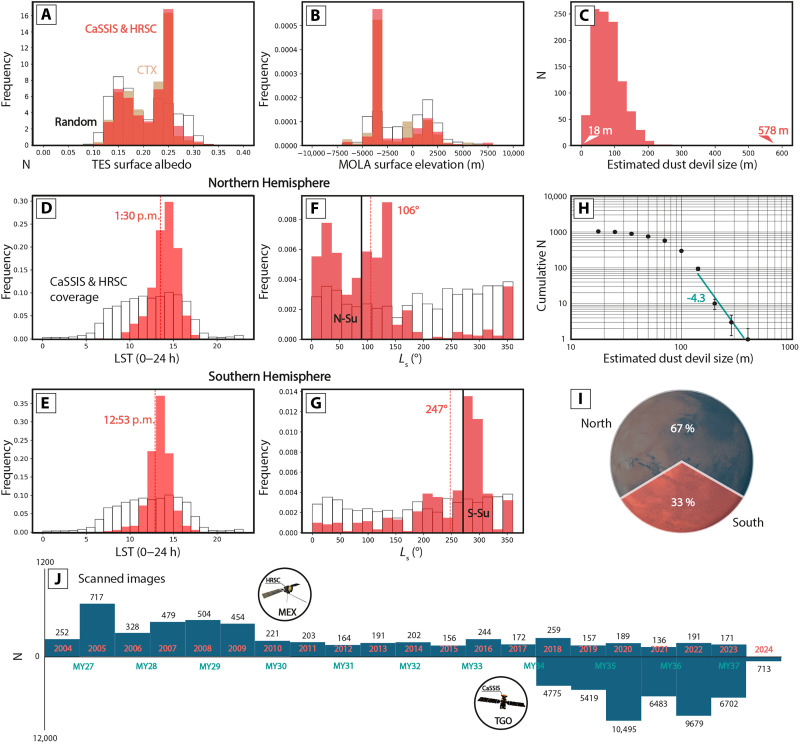
Dust devil morphometry, seasonality, and geostatistics. Distribution of CaSSIS and HRSC dust devil detections (red, min and max values indicated), CTX dust devil detections (tan), and 12,000 randomly placed points (black, constrained to 80°N to S) as a function of (**A**) TES surface albedo and (**B**) MOLA topographic elevation. (**C**) CaSSIS and HRSC estimated dust devil diameter distribution. (**D** to **G**) Distribution of CaSSIS and HRSC dust devils (red) and 1000 randomly placed points (black) as a function of *L*_s_ and LST, for the Northern and Southern Hemisphere; median values indicated, the *y* axis denotes probability density. (**H**) Size-frequency distribution (SFD; here, size represents the estimated vortex diameter) using √2 binning, power law fit, and exponent indicated by teal line and number, including √*n* error bars. The rollover between ~70 and ~100 m is likely caused by the limited spatial sampling of the CaSSIS and HRSC instruments. (**I**) Geographic distribution of all detected dust devils. (**J**) Number of scanned CaSSIS and HRSC images for MY 27 through MY 37.

Our dust devil detectors identify 384 (expert-verified) dust devils in CaSSIS images (in ~0.6% of the 39,475 images scanned) and 655 in HRSC images (in ~4% of the 5390 images scanned), all across Mars, between MYs 27 and 37 (104 dust devils/MY, Fig. 1, A to C). Only considering images with active dust devil (DD) vortices, the spatial density of detections is 0.002 DD/km^2^ for CaSSIS and 0.00003 DD/km^2^ for HRSC, which is likely controlled by the different spatial sampling of the two instruments (CaSSIS: 4 m/pixel; HRSC: >12 m/pixel) and the relative scarcity of larger dust devils (Fig. 2, C and H). Dust devil detections seem to be spatially colocated in the Northern Hemisphere, as dominated by the clusters in Amazonis and Elysium Planitiae as well as the lack of dust devils in Tharsis and Arabia Terra. In turn, dust devils appear to be more widespread in the Southern Hemisphere, with dust devil detections stretching over nearly the entire Southern Hemisphere (Fig. 1C).

Our data indicate that dust devils occur on topographically smooth (e.g., northern lowlands) as well as rough terrain (e.g., southern highlands). Overall, 67% of all detections are located in the north and 33% are located in the south. Our data show a large-scale spatial correlation with active dust devil vortex detections [e.g., (*56*–*59*)] as well as dust devil track observations (*59*) made in other orbital datasets, with local differences (Fig. 1C). The highest number of dust devils was detected in MY 37, and the lowest number was detected in MYs 31 and 33, which can likely be attributed to variations in the number of downlinked images and the migration of Mars Express orbit’s periapsis (Fig. 2J). We are able to derive migration measurements for 373 of the 1039 dust devil detections (~36%), 16 with CaSSIS stereo (4%), 298 with CaSSIS color fringe (80%, where each CaSSIS stereo measurement is complemented by a CaSSIS color fringe measurement), and 75 with HRSC stereo images (20%). The CaSSIS and HRSC dust devil migration dataset is openly available here: https://doi.org/10.48620/87803.

### Dust devil seasonality

Our observations confirm that dust devil occurrence is strongly controlled by the seasons (*58*), with a very rapid and distinct seasonal transition (Figs. 1C and 2, F and G, and fig. S2). We also confirm the seasonal, latitudinal band of dust devil occurrence between ~50° and ~60°S (southern summer) as recently identified by Conway *et al.* (*58*) in Mars Reconnaissance Orbiter (MRO) Context Imager (CTX) data (Fig. 1C). Our observations hint at a hiatus of dust devil activity between *L*_s_ ~200 and ~250° (solar longitude, late northern fall) across the entire Northern Hemisphere (Fig. 2F), as previously noted by researchers (*57*, *58*, *60*, *61*) using lander- and orbiter-derived measurements. This hiatus coincides with an atmosphere that is remarkably dust-free in late northern fall (*61*, *62*). We do not note a similarly expressed (and extensive) hiatus in the Southern Hemisphere, although southern dust devil activity is substantially reduced in southern winter as well (Fig. 2G).

### Dust devil diurnal distribution

Globally, the first dust devils are observed in the morning at ~8 a.m. and the last in the late afternoon at ~5 p.m., which slightly expands the time spans reported by landed missions like Spirit [9:30 a.m. to ~4:30 p.m. (*44*)] and Pathfinder (*63*), but matches more recent observations by Perseverance with detections at 5 and 5:30 p.m. (Fig. 2, D and E) (*29*, *64*). Globally, dust devil peak occurrence is between ~11 a.m. and ~2 p.m., with a slight difference between the Northern and Southern Hemisphere (~1:30 p.m. versus ~12:50 p.m.), agreeing with observations made by, e.g., Spirit (*44*) and Pathfinder (*65*), shifting earlier, regional, HRSC-derived estimates [~2 to ~3 p.m. (*60*)] closer to noon. Generally, there are between ~10 to ~32 times and between ~1.5 to ~4 times more dust devils at peak occurrence than at 10 a.m. and 3 p.m. (for the Northern and Southern Hemisphere), respectively, which helps explain the differences in dust devil vortex counts derived from data acquired by sun-synchronous orbiters with different revisit times such as the Mars Orbiter Camera on Mars Global Surveyor [~1 to ~3 p.m., ~2300 dust devils/MY identified (*57*)] and CTX on MRO [~3 to 3:30 p.m., ~1600 dust devils/MY identified (*58*)], acknowledging the substantial impact of the total number of downlinked images on those counts. Spatiotemporally coordinated observations by CaSSIS and HRSC in the Erebus Montes region (177°E, 35°N) indicate that the overall abundance of dust devils remains relatively constant from day to day, 0.002 DD/km^2^ (HRSC hq425_0000, 7 December 2024, ~1:30 p.m., *L*_s_ ~ 12°), 0.008 DD/km^2^ (CaSSIS MY38_031423_036, 8 December 2024, ~2:25 p.m.), and 0.003 DD/km^2^ (HRSC hq435_0000, 9 December 2024, ~2:20 p.m.), where the difference in density between CaSSIS and HRSC is likely caused by the difference in image pixel scale (~4 m versus ~25 m in this particular image sequence) (fig. S3).

### Dust devil morphometry and geostatistics

The diameters of dust devils range from an estimated ~18 to ~578 m, with a mean diameter of 82 m. Earlier, regional studies using orbital data derived dust devil diameters between ~50 and ~500 m (*60*, *66*, *67*), with a mean diameter of ~230 m, which agrees well with our data. The combination of CaSSIS and HRSC data provides high spatial sampling with an extensive spatial coverage (i.e., image footprint size), which enables the detection of both small and large dust devils (Fig.2, C and H). The CaSSIS- and HRSC-estimated size distribution is well described by a power law with a negative exponent of 4.3, which is smaller than earlier estimates based on orbital and in situ observations [−2 (*68*, *69*)]. This observation confirms earlier findings that very large dust devils are less common than small dust devils. Our data display a rollover between ~70 and ~100 m, which is likely caused by the limited spatial sampling of the used orbital data, as smaller dust devils have been observed by rovers in situ [e.g., (*69*)]. Our observations indicate that the dust devil diameter depends on LST and latitude, where larger dust devils occur at noon and in the afternoon, and more large dust devils occur at the mid-latitudes (~20° to ~40°N and ~40° to ~65°S, fig. S4, B and D). We expect the latitudinal difference between the enhanced occurrence of large dust devils in the north and south (~20° to 40°N versus ~40° to ~65°S) to be predominantly controlled by the northern dust devil hotspots and monitoring sites (centered at ~30°N), such as Amazonis, as well as the southern, seasonal dust devil band (centered at ~50°S).

Dust devils appear in the same TES (Thermal Emission Spectrometer) albedo terrain and at the same MOLA (Mars Orbiter Laser Altimeter) elevations as identified globally by Conway *et al.* (*58*) using CTX data (Fig. 2, A and B), providing further evidence that dust devils can occur at a very wide range of altitudes and atmospheric pressures. For example, HRSC and CTX imaged dust devils in the vicinity of the Elysium Mons caldera at elevations of ~6700 to ~10,800 m, and CTX captured an active dust devil vortex in the vicinity of the Olympus Mons caldera at an elevation of ~21,000 m (*58*), highlighting that dust devils can occur at extremely high altitudes.

### Dust devil migration

**Fig. 3.**
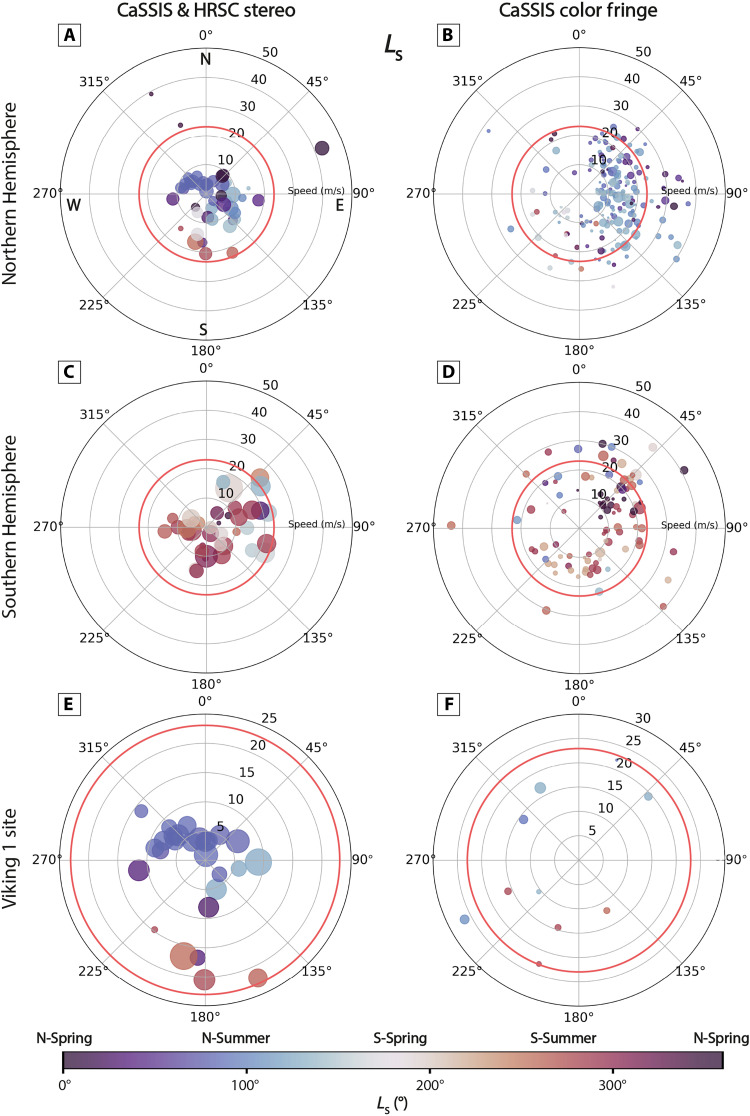
Dust devil migration—seasons. Polar migration plots for CaSSIS and HRSC stereo-measured (left) as well as CaSSIS color fringe–measured (right) dust devils, for the Northern Hemisphere (**A** and **B**), Southern Hemisphere (**C** and **D**), and the wider Viking 1 landing site (**E** and **F**). Radius indicates migration velocity, theta angle indicates migration azimuth (0° North, 90° East, and so on), and shape size indicates estimated dust devil diameter (small to large). Shape color represents *L*_s_ of occurrence. The static threshold range (as applied in this work) is approximated by solid red circles (wind velocity at an altitude of 10 m above the surface). Viking 1 data taken from (*116*).

**Fig. 4.**
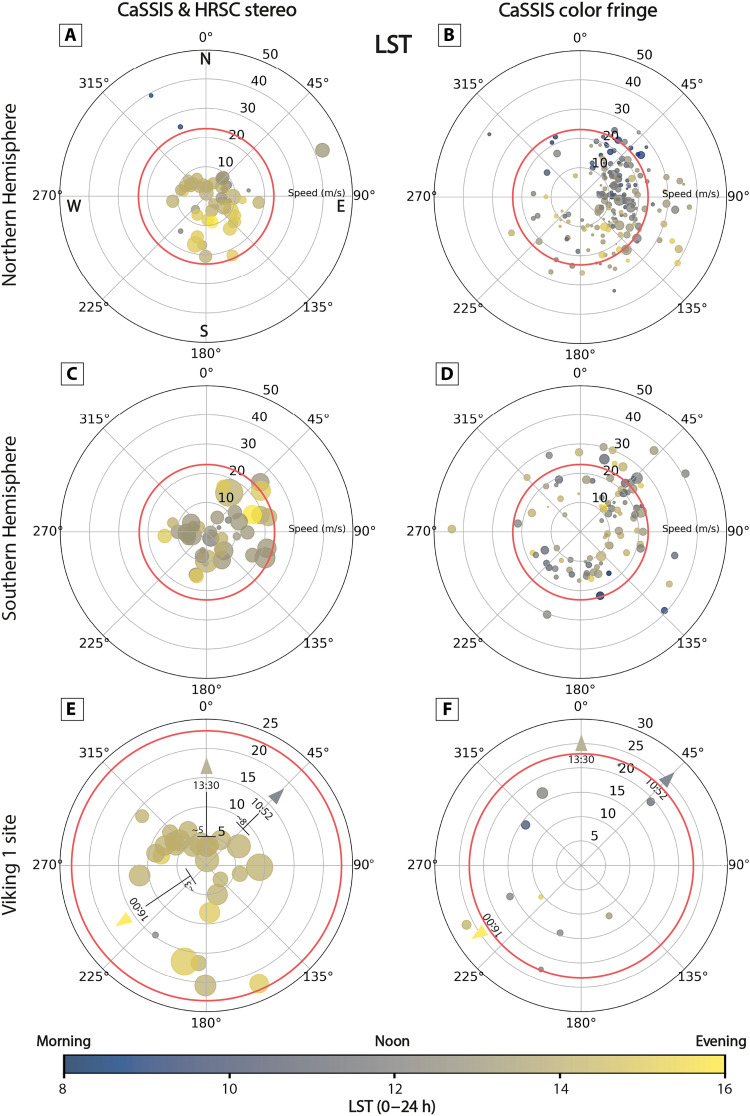
Dust devil migration—local solar time. Polar migration plots for CaSSIS and HRSC stereo-measured (left) and CaSSIS color fringe–measured (right) dust devils, for the Northern Hemisphere (**A** and **B**), Southern Hemisphere (**C** and **D**), and the wider Viking 1 landing site (**E** and **F**). Radius indicates migration velocity, theta angle indicates migration azimuth (0° North, 90° East, and so on), and shape size indicates estimated dust devil diameter (small to large). Shape color represents the LST of occurrence. The static threshold range (as applied in this work) is approximated by solid red circles (wind velocity at an altitude of 10 m above the surface). The average wind velocities and directions as measured by Viking 1 in the morning, noon, and afternoon are indicated in (E) and (F) by triangles (color indicates LST). Viking 1 data taken from (*116*).

In the northern low latitudes (<30°N), dust devils tend to migrate pole-ward in northern summer (~83 to ~50%, in stereo and color fringe data, respectively, *L*_s_ 45° to 135°) and equator-ward in southern summer (~100%, in color fringe data, *L*_s_ 225° to 315°). In the southern low latitudes (<30°S), that trend is reversed and distinct in northern summer (*L*_s_ 45° to 135°), with ~100 to ~89% of dust devils migrating equator-ward (in stereo and color fringe data, respectively), although less distinct in southern summer (*L*_s_ 225° to 315°), with ~67 to ~28% of dust devils moving pole-ward (in stereo and color fringe data, respectively). Overall, these observations of the migration of low-latitude dust devils are consistent with the seasonal meridional wind direction. In the northern low latitudes, stereo measurements indicate that equator-ward–moving dust devils (southern summer) are a factor of ~3 faster than pole-ward–moving dust devils (northern summer), on average, although that trend is not reflected in the color fringe measurements (Figs. 3 and 4).

In the mid-latitudes (30° to 60°N and S), dust devil abundance is strongly seasonally controlled in both hemispheres. We do not observe any pole- or equator-ward migration trend, but the vast majority of dust devils migrate eastward, 77 to 89% in the north and 53 to 73% in the south, in stereo and color fringe data, respectively. In the southern mid-latitudes, eastward-migrating dust devils feature slightly higher velocities than westward-migrating dust devils, a factor of ~1.5, on average, in stereo data, respectively, although that trend is not reflected in the color fringe measurements or in the Northern Hemisphere (Figs. 3 and 4). Generally, dust devil migration seems to be more strongly controlled by the seasons and LST in the Northern Hemisphere than in the Southern Hemisphere, most probably because of topographic effects (Figs. 3 and 4). We note that the second to minute timescale migration dynamics of dust devils, as observed by CaSSIS and HRSC, might not always reflect the global-scale circulation or even regional wind direction, due to topographic and other effects.

Dust devil movement and the associated winds can be as fast as ~44 m/s, in all seasons, with a mean velocity of ~11 and ~18 m/s as derived by stereo and color fringe measurements, respectively, similar to regional measurements derived by Stanzel *et al.* (*60*, *67*) who report a mean velocity of ~23 m/s (Figs. 3 and 4 and fig. S5). Note that the term “velocity” refers to the straight-line (“lateral”) velocity of dust devils and the associated wind field throughout this manuscript (versus, e.g., the rotational velocity). We expect our velocity measurements to be accurate to ±~4 m/s for all CaSSIS color fringe, and to ±~0.2 and ±~1.3 m/s for all CaSSIS and HRSC stereo measurements, respectively. The observed peak velocity is substantially faster than peak (nonvortical) wind velocities measured at any rover/landing site, such as the InSight [~31 m/s (*49*)], Perseverance [~32 m/s (*29*)], and Viking 1 and 2 landing sites [~12 and ~23 m/s (*48*, *70*)].

Our global observations confirm that martian dust devils move substantially faster than on Earth, a factor of ~2.5 (*27*, *44*, *71*–*73*). Still, about 78 and 41% of the dust devils in the CaSSIS and HRSC dataset (for stereo and color fringe measurements, respectively) remain at velocities below 15 m/s, which is the peak velocity recorded for dust devils on Earth [fig. S5 (*72*)]. We acknowledge that Stanzel *et al.* (*60*) report two dust devils that exceed our peak velocity of ~44 m/s, with velocities of ~54 and ~59 m/s (± 7 m/s) (HRSC images h2035_0000 and h2046_0000), but we are not able to reproduce those measurements. Instead, we measure peak velocities of ~42 and ~30 m/s (± 1.5 m/s) in the respective images. Notably, all colocated dust devils in the respective HRSC images (h2035_0000 and h2046_0000) feature velocities well below 40 m/s (average velocities of ~27 and ~26 m/s, respectively). We attribute the two excessive dust devil velocities measured by Stanzel *et al.* (*60*) to the limited accuracy and spatial resolution of the HRSC data used [level 3 in (*60*) versus level 5 as used in our study].

Globally, dust devil velocity does not appear to depend on the longitude, latitude, *L*_s_, or estimated diameter, agreeing with earlier, regional observations (*60*) as well as terrestrial observations (*71*), although very slow dust devils were only identified at mid-latitudes. We note a slight dependency of velocity on LST, where both the highest and lowest recorded velocities occur at noon (fig. S2I). Similarly, (globally) dust devil migration azimuth does not depend on longitude, latitude, or diameter (fig. S2D). We observe a dependency of dust devil azimuth on LST, with the widest variability of azimuths at noon and in the early afternoon, and the smallest variability in the morning and late afternoon (fig. S2J).

**Fig. 5.**
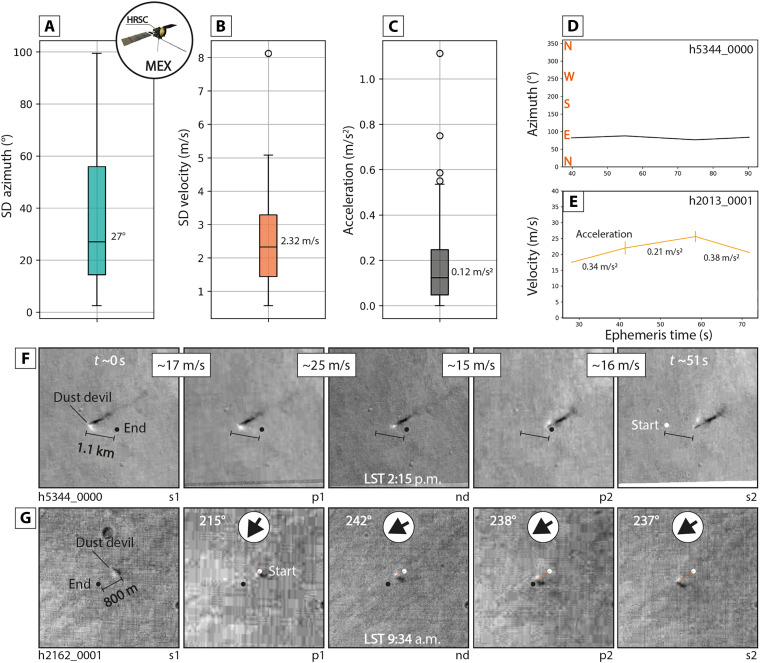
Dust devil short-term migration behavior. Box-and-whisker plots of (**A**) SD (standard deviation) of all HRSC-observed dust devil azimuth, (**B**) SD of all observed dust devil velocities, and (**C**) SD of observed dust devil acceleration/deceleration. Examples of dust devil azimuth (**D**) and velocity (**E**) for two HRSC-observed dust devils; acceleration measurements indicated in (E). HRSC image examples of short-term (~51 s) dust devil velocity (**F**) and azimuth (**G**) variations; azimuth indicated by arrows in (G). Image credit: ESA/DLR/FU Berlin CC -BY-SA 3.0 (https://creativecommons.org/licenses/by-sa/3.0/deed.en) IGO; the shown images were cropped from the original HRSC images.

On the second to minute timescale, dust devil velocity and azimuth can vary around ~2.3 m/s and ~27° (median), and individual dust devils can change their velocities on the order of ~0.1 m/s^2^ (median) (Fig. 5, A to C). This implies that even dust devils and associated, nonvortical near-surface winds with intermediate velocities might only need a few seconds to accelerate to velocities that suffice to initiate particle saltation and atmospheric dust injection. Notably, the observed azimuth variations agree very well with measurements of the orientation of dust devil tracks, such as performed around InSight [SD of azimuth: 25° to 37° (*74*)], and with manual observations made using CaSSIS images (fig. S6). In addition, we observe a distinct dependency of dust devil azimuth variations on dust devil velocity (fig. S7), as previously recognized in (*71*, *75*) for terrestrial dust devils. This relation might enable a utilization of dust devil track “linearity” to infer estimates of dust devil—and ambient wind field—velocities. We note that among the 373 dust devils with migration measurements, we observe one possible example of the instantaneous, minute-timescale formation of a dust devil (fig. S8).

Notably, a regional population of dust devils distributed over hundreds of kilometers, as occasionally captured by individual HRSC images (e.g., hq425_0000), can exhibit velocity variations between ~4 and ~8 m/s and azimuth variations of up to ~75°, highlighting how our measurements of azimuth might not fully reflect the azimuth of the global-scale, regional, and/or local wind field. In addition, coordinated, short-term, high-cadence (~3 Earth days, 1 CaSSIS/HRSC images per Earth day) observations of a dust devil hotspot in the Erebus Montes (177°E, 35°N) show that dust devil migration (and wind field) dynamics can drastically change on day-to-day timescales, with azimuth changes as large as ~120° over the course of 3 days (fig. S3). On the other hand, long-term (~16 Earth days, 0.4 CaSSIS images per Earth day) observations of a dust devil hotspot in Amazonis Planitia (164°W, 25°N) suggest that dust devil azimuths and velocities during the daytime convective period can be very similar from sol to sol over timescales on the order of a week or more (e.g., changes of less than ~3 m s^−1^ day^−1^, fig. S9).

### Wind shear velocities and wind stresses

Wind shear velocity—also referred to as drag velocity and friction velocity—is the most widely used measure to describe the static threshold—and with it, active dust lifting conditions—but does not consider the atmospheric density, which can vary substantially in space and time and has a substantial effect on the effective stress induced by the wind. Wind stress represents a more physical descriptor of dust lifting conditions, as it is a function of the atmospheric density and the wind shear velocity [e.g., (*29*)]. Direct measurements of martian wind stress do not exist, but estimates can be derived from wind shear measurements and model estimates of atmospheric density.

**Fig. 6.**
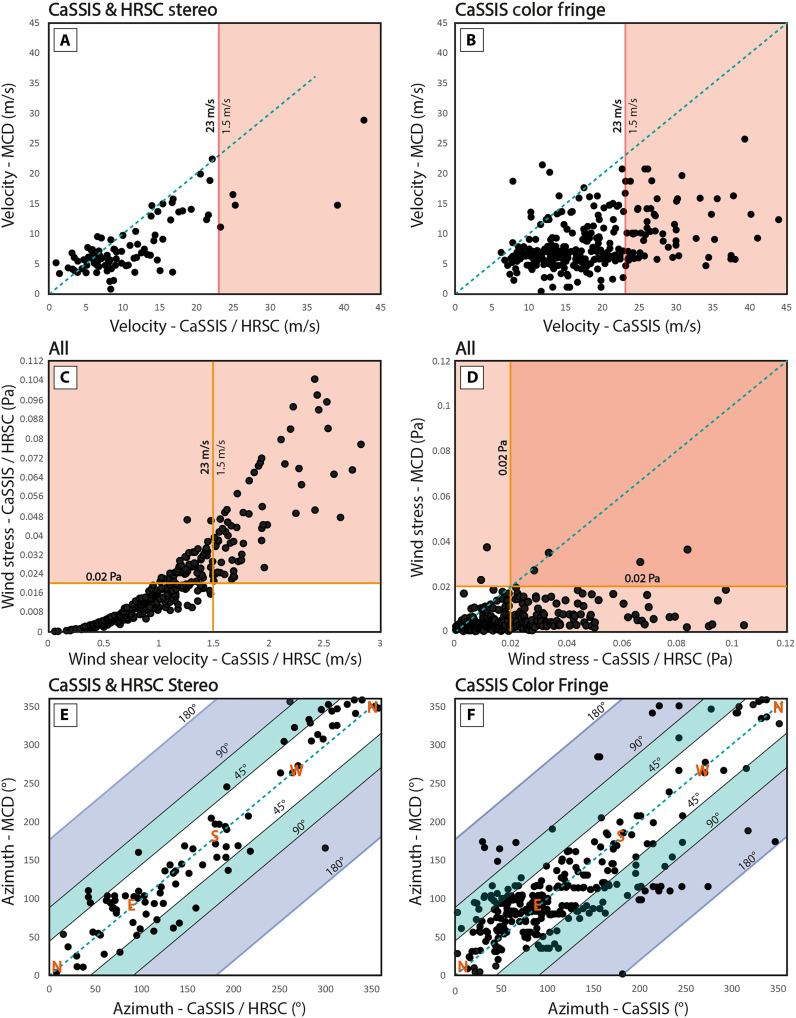
Comparison of CaSSIS and HRSC measurements with MCD global-scale predictions. Comparison of CaSSIS stereo and HRSC stereo-observed (**A**) and CaSSIS fringe-observed (**B**) dust devil migration/nonvortical wind field velocities with values predicted by the MCD at a height of 10 m above the surface; (**C**) comparison of CaSSIS/HRSC-derived wind stress wind stress and wind shear velocity; (**D**) comparison of CaSSIS/HRSC-derived wind stress and MCD-predicted wind stress. Wind stress threshold of 0.02 Pa (red or orange) is indicated in (C) and (D) and a translation to wind velocity/shear velocity is indicated in (A and B) circles (at an altitude of 10 m above the surface). Comparison of CaSSIS stereo and HRSC stereo-observed (**E**) and CaSSIS fringe-observed (**F**) dust devil migration/wind field azimuths with values predicted by the MCD at a height of 10 m above the surface; azimuth deviations of >45° (teal) and >90° (violet) are indicated.

For vortical processes, wind shear velocities beyond ~1 m/s have been associated with active dust lifting, translating to wind stress values beyond ~0.015 Pa for an average atmospheric density of 0.014 kg/m^3^. In situ measurements of shear velocities associated with nonvortical processes that caused active dust lifting are missing, but a few past missions provide lower bounds: For example, Sagan *et al.* (*76*) report wind shear “gusts” in excess of ~1.4 m/s that were able to displace sand and dust grains, and Newman *et al.* (*29*) report straight-line wind velocities of ~15 m/s (at ~1.5 m above the surface) and converted wind shear in excess of ~1.5 m/s that caused active, large-scale dust lifting, translating to a wind stress of ~0.02 Pa assuming an average atmospheric density. For this work, we use a wind stress threshold of 0.02 Pa as a first-order descriptor of active dust sourcing conditions for nonvortical, straight-line winds (Fig. 6C), noting that the threshold might potentially be substantially higher or lower [e.g., (*51*)]. Depending on the atmospheric density, this wind stress threshold converts to a wind velocity of ~23 m/s at an altitude of ~10 m above the surface, as sampled by the observed dust devil population, assuming an average aerodynamic surface roughness of 0.02 m and a convectively stable atmosphere.

Overall, ~11 and ~39% of the observed dust devils and their associated wind fields exceed a wind stress of 0.02 Pa, i.e., the threshold as defined above, in stereo and color fringe data, respectively. This implies that between ~11 and ~39% of all dust devils observed by CaSSIS and HRSC (with velocity measurements, for stereo and color fringe measurements, respectively) present a dynamical state of the lowermost atmosphere that likely provides conditions suitable for the active lifting and atmospheric injection of dust through nonvortical, near-surface winds. For comparison, assuming a lower, less conservative wind stress threshold such as 0.008 Pa [as used by Newman *et al.* (*29*) for parts of their analysis] would lead to ~71% of dust devils and associated winds exceeding the wind stress threshold. The fastest dust devils and near-surface winds observed by CaSSIS and HRSC convert to shear velocities and wind stresses of up to ~2.8 m/s and ~0.1 Pa, respectively, which is sufficiently strong to initiate the saltation of a wide range of particle sizes between ~30 and 800 μm, way beyond the ~100-μm particle size range that is most susceptible to wind shear (*36*, *49*). In situ observations imply that such strong, dust-lifting winds are unlikely to persist continuously for extended periods of time [e.g., (*29*)], but the CaSSIS and HRSC observations show that strong winds can (i) persist—at least—on timescales of approximately minutes and (ii) recur on a daily, weekly, and even monthly basis (figs. S3 and S9).

### Comparison with MCD outputs

The ambient (nonvortical) “background” wind velocities of the lowermost atmosphere (at 10 m above the surface) as predicted by the MCD on global scales systematically underestimate the lateral dust devil and near-surface wind velocities measured in CaSSIS and HRSC data (in ~76 and ~96% of cases) by ~3 and ~10 m/s, on average, for CaSSIS and HRSC stereo and CaSSIS color fringe measurements, respectively (Fig. 6, A and B). Similarly, the wind stress predicted by the MCD systematically underestimates the wind stress values derived from the combination of CaSSIS and HRSC dust devil velocity measurements and MCD-predicted atmospheric density (Fig. 6D). Only 6 dust devils and their associated winds would exceed our assumed wind stress threshold per the MCD predictions (~2% of full dataset), while 124 dust devils and their associated wind fields exceed the wind stress threshold per the CaSSIS and HRSC measurements (~33% of full dataset). Overall, the derived dust devil azimuths partially agree with the MCD-predicted values. We note that in 23 and 30% of the cases, the MCD azimuth is more than 45° off, and in 2 and 8% of the cases, it is more than 90° off, for stereo and color fringe measurements, respectively (Fig. 6, E and F). The MCD predictions are never more than ~133° off for CaSSIS/HRSC stereo measurements, but can be up to 180° off for CaSSIS color fringe measurements (*n* = 2). For (fast) dust devils that exceed velocities of 23 m/s, the MCD-predicted azimuth has been partially representative, with a mean azimuth difference of 16° and 51°, and a maximum difference of 28° and 104°, for stereo and color fringe measurements, respectively.

The difference between our migration measurements and MCD-derived, global-scale predictions (velocity and azimuth, per dust devil) does not depend on *L*_s_ but partially on LST, where the largest differences occur around noon and in the early afternoon, which are likely connected to unresolved topographic processes and the generally wider variation of wind velocities and azimuths at that time of the day (figs. S2 and S4). Similarly, the differences between our measurements and the MCD predictions (difference velocity versus difference azimuth) do not show a trend, suggesting that there is no apparent relation between velocity and azimuth differences (fig. S2). Overall, our observations (as expected) imply that the MCD’s global-scale predictions do not fully capture the complete range of the meso-/sub–regional-scale dynamics of the lowermost martian atmosphere as captured by our observations of dust devil dynamics and, with it, details about the locations and timing of the sourcing and atmospheric injection of dust (Fig. 6). Yet, there is a substantial level of agreement between MCD-predicted and dust devil–derived wind field azimuth. The CaSSIS and HRSC dust devil migration data can serve as ground-truth data to further validate and fine-tune the MCD as well as other GCMs and meso-scale atmospheric models in the future [e.g., (*77*)]. We present a summary of all measurements derived in this study in table S1.

### Dust lifting and atmospheric injection

**Fig. 7.**
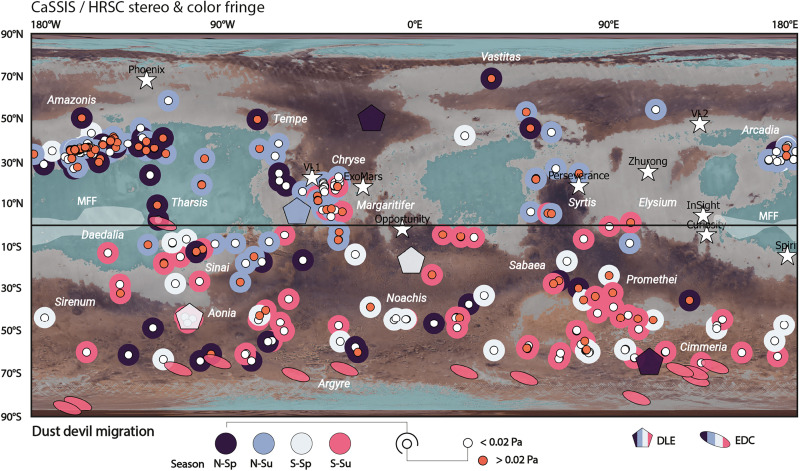
Spatiotemporal comparison of CaSSIS and HRSC wind stress estimates with MCD regional predictions and dust source regions. Global map showcasing where and when (i.e., season) CaSSIS/HRSC stereo- and CaSSIS color fringe–derived dust devils and wind fields exceed the assumed wind stress threshold (innermost shape, orange >0.02 Pa wind stress, white <0.02 Pa); the outermost shape indicates the season of occurrence. Locations and seasons of occurrence of HRSC-observed dust lifting events (DLE, pentagons) and elongated dust clouds (EDC, ellipses) indicated (*80*). Colorized TES albedo indicates dusty (gray) and very dusty (fine dust, teal) regions. Locations of landed/future missions (white stars) and the Medusae Fossae Formation (MFF) indicated. Viking color mosaic in the background.

Our observations of dust devil and wind field dynamics prove that nonvortical gusts and straight-line winds in excess of the wind stress threshold are abundant on Mars and occur at multiple locations across the planet and at multiple times over all seasons (Fig. 7). In addition, we observe that a substantial fraction of the “fast” dust devils and their “strong” associated wind fields that exceed the assumed wind stress threshold and whose wind stress values are underestimated by the MCD are located in dust-bearing regions, including regions with particularly fine dust [per TES and, e.g., (*78*, *79*)] (Fig. 7). This observation implies that strong, nonvortical winds are likely to inject a substantial amount of dust into Mars’ atmosphere, and substantially more than previously anticipated. In this way, our dataset marks regions and times of general, active dust sourcing through nonvortical processes—although it remains unclear how persistent these strong winds and dust-lifting conditions are beyond timescales of minutes (Fig. 7).

**Fig. 8.**
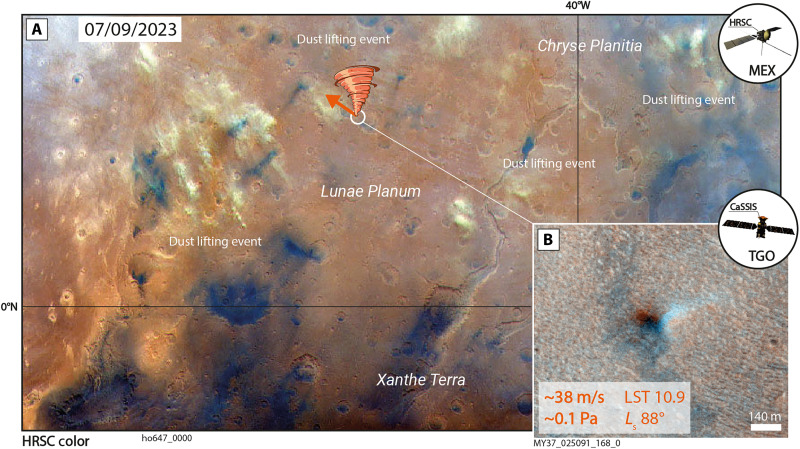
Spatiotemporal colocation of a fast dust devil and strong nonvortical, near-surface wind and a large-scale dust lifting event in Lunae Planum. (**A**) Spatiotemporal colocation (orange shape, LST, *L*_s_, velocity, and azimuth indicated) of a fast CaSSIS dust devil and a large-scale dust lifting event (beige clouds) observed by HRSC on 9 July 2023 in the wider Lunae Planum area. Arrow indicates dust devil azimuth (dust devil is located at the tip of the orange vortex). (**B**) CaSSIS zoom-in image of dust devil. Image credit: ESA/TGO/CaSSIS CC -BY-SA 3.0 (https://creativecommons.org/licenses/by-sa/3.0/deed.en) IGO, ESA/DLR/FU Berlin CC -BY-SA 3.0 (https://creativecommons.org/licenses/by-sa/3.0/deed.en) IGO; the shown images were cropped from the original CaSSIS and HRSC images.

We identify five fast-moving dust devils and strong wind gusts that occur in the same region and season, i.e., *L*_s_ ±15° and ±10° longitude/latitude, as HRSC observations of larger-scale dust lifting events [per (*80*), covering MY 36 to MY 37, 2021 to 2023] (Fig. 7). The spatiotemporal scarcity and heterogeneity of CaSSIS, HRSC, and Mars Color Imager data [MARCI on MRO (*81*)] makes it difficult to correlate fast dust devils and strong gusts with larger-scale atmospheric features in space and absolute time, but we identify one dust devil and associated wind field with a velocity of ~38 m/s (wind stress ~0.1 Pa) in the wider Lunae Planum area (on 9 July 2023), where a large-scale dust lifting event occurred on the same day (Fig. 8). Similarly, we observe overlap (spatially and in absolute time) of three fast dust devils and strong wind gusts (>22 m/s, >0.03 Pa) with the MY 28 PEDE and cataloged dust storm events G03 and G04 in MY 30 (September and October 2010) (*82*) (fig. S10). There exists no consistent dust storm catalog for the period MY 27 to 37, but we identify six additional dust devils with velocities >23 m/s (>0.03 Pa) that seem to be associated (±10° longitude/latitude) with MARCI-scale dust lifting events, dust clouds, haze, or other dust-related atmospheric activity (fig. S11). Notably, we also identify examples of fast-moving dust devils and wind gusts that do not seem to be associated with other dust-related atmospheric activity (fig. S12).

We estimate the amount of dust lifted (vortically) by our dust devil catalog following the workflows developed and used by earlier studies (*44*, *60*, *83*), using estimates of dust devil lifetime and travel distance that are based on our more representative (CaSSIS- and HRSC-derived) measurements of dust devil dimensions that include dust devils identified globally and over a substantially wider range of MY and LST—ignoring any measures of velocity and the contribution of (nonvortical) near-surface winds. Our database suggests that, on average, a dust devil lifts between 3.2 and 79 (north) and between 2.9 and 72 (south) tons of dust, and between 0.008 and 0.21 kg/m^2^ of dust (north and south, for an average area of 0.28 km^2^ modified per dust devil). Overall, our computations suggest that the observed dust devils lifted—in total—between 2.2 × 10^3^ and 5.5 × 10^4^ (north) and between 1 × 10^3^ and 2.5 × 10^4^ (south) tons of dust into the martian atmosphere between MY 27 and MY 37 [using two different dust flux estimates derived by Greeley *et al.* (*44*) and Metzger *et al.* (*83*), where “flux” describes the dust mass lifted per area per time], i.e., between 2.2 × 10^2^ and 5.5 × 10^3^ and between 1 × 10^2^ and 2.5 × 10^3^ tons/MY (north and south, respectively), which is substantially less than the mass moved by a typical global dust storm [4.3 × 10^8^ tons (*84*)]. Our estimates represent a lower bound, as our database only contains a very small fraction of all dust devils that were active in the covered time period.

We address the limited spatiotemporal coverage of our CaSSIS- and HRSC-derived catalog by using global dust devil density indices estimated by Whelley and Greeley (*85*) (for the Northern and Southern Hemisphere), including estimates of dust devil lifetime based on the size-frequency distribution (SFD) of the CaSSIS- and HRSC-detected population, but without including its dust devil counts. We estimate dust lifting rates between 1.9 × 10^−4^ and 4.7 × 10^−3^ (north) and between 1.7 × 10^−3^ and 4.3 × 10^−2^ kg m^−2^ MY^−1^ (south). All of the above vortical estimates do not consider the presumably substantial contribution of the strong, nonvortical near-surface winds associated with 11 to 39% of the identified dust devils.

## DISCUSSION

### Limitations of the data and methods

Despite the substantial spatial and temporal extent of the dust devil migration dataset (global coverage, MY 27 to 37, Fig. 2J), the HRSC and CaSSIS data used here provide only snapshots in space and time, as both instruments can only cover a minimal fraction of the day-side martian surface at a given point in time, and only for a short amount of time (second to minute timescale). This means that CaSSIS and HRSC are not able to quantify the persistence of strong winds and dust-lifting conditions beyond the approximately minute timescale. Similarly, MARCI data are not always available and might contain large spatial gaps (Fig. 8 and figs. S10 to S12). We note that fast near-surface winds might not always co-occur with dust devils and, thus, might regularly be invisible to CaSSIS and HRSC. As of August 2025, there is no consistent, global dust storm, dust cloud, or dust lifting event catalog; detailed dust storm information is only available for MY 24 to 32 (*82*), with limited temporal overlap with HRSC (and none with CaSSIS). In addition, the test set performance of the deployed deep learning detectors indicates that our dust devil catalog might miss about ~20 to ~30% of all dust devils imaged by CaSSIS and HRSC (fig. S14).

Because of the limited spatial resolution of CaSSIS and HRSC, this work can only consider dust devils (i.e., dust-bearing vortices) larger than ~18 m (for CaSSIS) and larger than ~60 m across (for HRSC), which is not the same population that has been observed by landed missions [e.g., (*42*, *49*, *86*)]. Our estimates of dust devil diameter are carefully statistically calibrated following an approach recently demonstrated by Conway *et al.* (*58*), but are based on the rectangular bounding boxes output by the two separately trained and deployed CaSSIS and HRSC detectors—and thus are likely to output slightly different populations of dust devil size estimates, amplified by the substantial difference in pixel scale across the CaSSIS and HRSC datasets (fig. S15). At the same time, CaSSIS might not be able to properly capture and recognize extremely large dust devils in their footprints (>~500-m-scale dust devils), due to field-of-view limitations, or might confuse them with large clouds due to a lack of spatial context.

Our measures of dust devil migration depend on the accuracy of the user-determined position of each dust devil in a given image, and thus on the visual perception of the morphological center or color fringes of each dust devil, which is not always obvious (Fig. 4 and fig. S16). The errors that are introduced by this uncertainty could influence the accuracy of the resulting measurements, particularly for the measurements derived in CaSSIS color fringe data, which features the smallest temporal differences (~1 s) and thus the smallest relative displacements. Despite the good agreement of azimuth and velocity measurements derived by the CaSSIS stereo and CaSSIS color fringe approaches in our cross-validation dataset (fig. S16), the uncertainties associated with the color fringe method, such as caused by, for example, turbulent vortices that mimic rapid lateral movements (figs. S1 and S16), which might potentially be underrepresented in the validation data, are more likely to make the respective measurements less reliable, particularly the velocity measurements. These uncertainties might explain the increased number of fast dust devils in the CaSSIS color fringe data (versus the CaSSIS stereo and HRSC stereo data, e.g., Fig. 6, A and B). The CaSSIS color fringe approach enables the analysis of a factor of ~4 to ~17 more dust devils than enabled by the HRSC and CaSSIS stereo data alone (*n* = 75 and *n* = 16 versus *n* = 298), which renders the color fringe–derived results generally more representative of the overall, global, spatiotemporal behavior of dust devils. All of the described data and technical limitations might skew the overall distribution of the statistical and morphometric properties as reported in this work, as well as their comparison with other studies, and might affect our interpretations.

Last, as fundamental assumptions of this work, we follow (*27*, *60*, *65*, *67*, *71*, *87*) in assuming that dust devils (i) travel with the lateral (nonvortical), ambient wind field—not faster or slower than the ambient wind field—and (ii) properly represent the lateral wind field velocities at an altitude of 10 m above the surface. We underpin these assumptions with qualitative and quantitative evidence of dust devil vortices moving with the same velocity and azimuth as spatiotemporally colocated, diffuse (dust) clouds as an approximation of the behavior of the ambient wind field (fig. S19), but note that both assumptions might still oversimplify the actual behavior of the near-surface wind field and thus could affect our calculations of shear velocities and wind stresses. Last, we assume that our observations of dust devil and wind velocity are representative of the actual distribution of wind velocities across Mars. We also note that the utilization of MCD-predicted atmospheric density values might introduce an error in our calculation of wind stress.

### Atmospheric dust sourcing on Mars

Despite those limitations, our data show that there are a substantial number of fast dust devils and, thus, strong, nonvortical near-surface winds all across Mars that seemingly exceed wind stress values of 0.02 Pa (Fig. 6). A substantial fraction of those dust devils and winds are located in regions that were previously proposed as global dust sources based on MARCI-scale observations and GCM outputs, specifically northern Tharsis, Amazonis, Chryse, Syrtis, Margaritifer, Hellas, Aonia, Arcadia, Promethei, Sabaea, Cimmeria, and Aonia (*3*, *7*, *14*), including the MFF, which recently has been proposed as Mars’ single-largest dust source (*21*) (Fig. 7). Our observations provide direct observational evidence that all of those regions are active dust source regions, although it remains unclear when and over what time periods exactly. Our data suggest substantially higher wind stresses and velocities than currently anticipated and modeled by the MCD in all of those regions, likely because of the MCD’s (and other models’) coarse spatial gridding (Fig. 7) that predominantly captures global-scale and large-scale regional circulations.

We note that our data suggest a more prominent role of two potential dust source regions that have been largely unrecognized in the literature: Daedalia Planum (predominantly active in northern spring and summer) as well as Sinai Planum (predominantly active in southern spring and summer) and the Northern Hemisphere as a dust source in general (Fig. 7). The lack of (fast) active dust devil vortices and strong wind gusts in Arabia and large parts of Elysium might suggest that those regions have been dust sinks over the past MYs, and possibly longer. Notably, a hiatus of dust devil activity, such as observed by CaSSIS and HRSC in the Northern Hemisphere between *L*_s_ 200° and 250° (Fig. 2), usually leads to a distinctively dust-free atmosphere [as reported by, e.g., Wolkenberg *et al.* (*61*, *62*)], underlining the importance of dust devils and near-surface winds for the sourcing and atmospheric injection of dust. Our observations confirm earlier hypotheses that high ambient wind velocities do not generally suppress dust devil formation (fig. S5) (*60*, *88*, *89*).

A certain fraction of the identified dust devils and nonvortical gusts with wind stresses beyond 0.02 Pa can be associated with previously classified dust lifting events/storms (*80*, *82*) or visually associated with apparent, MARCI-scale dust lifting events, storms, or atmospheric haze (Figs. 7 and 8 and figs. S9 to S12). Those observations extend the recent in situ observations of local dust lifting events driven by near-surface winds (*29*) and support the hypothesis that strong winds, dust devil activity, dust lifting events, and—potentially—larger dust storms are directly connected, for example, as part of larger convection cells. It remains unclear whether fast-moving dust devils and the associated strong wind gusts indirectly [through continuous redistribution of dust (*2*, *56*)] or directly cause or lead to larger-scale dust lifting events and storms [as recently suggested in (*90*, *91*)], or whether they merely occur in the onset or wake of passing storm fronts as previously concluded in, for example, (*57*, *60*, *92*). If smaller-scale fast dust devils and strong nonvortical near-surface winds were confirmed as a driver—or indicator—of larger-scale dust lifting events and storms, then systematic, frequent observations of dust devil abundance and migration patterns could be used to anticipate and predict larger-scale dust lifting events and—potentially—storms. The presented CaSSIS and HRSC dataset represents a starting point for future, more systematic investigations of the potential spatiotemporal relations between wind gusts, dust devils, lifting events, and storms.

Our estimates of dust lifting rates—based on estimates of regional dust devil density by Whelley and Greeley (*85*) and ignoring lateral velocity and (nonvortical) near-surface winds—are a factor of ~5 lower than previously estimated in (*60*, *66*), which is most likely attributed to our refined and likely more accurate measurements of dust devil diameter as well as likely more representative assumptions about dust devil lifetime and travel distance. We note that our estimated dust lifting rates still indicate that dust devils with diameters between ~18 and ~500 m might contribute a substantial fraction of dust to the atmospheric circulation and global dust settling rate [2 × 10^−2^ kg m^−2^ MY^−1^, (*1*)], i.e., about ~9% [assuming an (*44*) dust flux]—or might entirely account for it, assuming a higher (*83*) dust flux, as was proposed by earlier studies [e.g., (*3*, *60*, *66*)]. However, we point out that the underlying dust devil densities, dust fluxes, and relations between dust devil lifetime and travel distance were derived for specific regions on Mars and suffer from various, substantial limitations.

Regardless of the reliability of the above estimates, these deliberations add further weight to the finding by Reiss *et al.* (*66*) that large dust devils in particular contribute substantially to the overall injected dust budget, because they modify exponentially larger fractions of the surface, are active for longer periods of time, and thus lift more dust (and to higher altitudes). However, complementary to previous studies (*66*, *68*, *69*), the CaSSIS and HRSC dust devil SFD suggests that very large dust devils are relatively rare (Fig. 2, C and H); yet, our observations also suggest that large dust devils occur not only in a few constrained regions but all over Mars, particularly at the mid-latitudes, further highlighting their seemingly substantial contribution to the overall atmospheric dust budget (fig. S2).

All of the vortical process–based estimates do not yet consider the presumably substantial effect of the (systematically underestimated) nonvortical near-surface winds on dust sourcing, despite the decisive role that has been attributed to near-surface winds on Earth [e.g., (*33*, *93*–*95*)]. CaSSIS and HRSC observations strongly suggest that strong, nonvortical near-surface winds are abundant on Mars and play a crucial role in atmospheric dust sourcing, but do not allow for a quantitative estimate of lifted volumes, rates, and dust-lifting durations. Future efforts need to combine our observations with GCMs and meso-scale atmospheric modeling to better characterize the role of nonvortical winds in the martian dust cycle.

### The role of dust devils and near-surface winds in shaping the surface of Mars

Our observations add further weight to the suggestion by McEwen *et al.* (*10*) and Bickel and Valantinas (*12*) that dust devils and strong, nonvortical wind gusts could play a role in (repeatedly) triggering RSL during southern summer (~*L*_s_ 270°), as suggested by their spatiotemporal overlap: Dust devils consistently occur at RSL locations, over all studied MY, and predominantly in southern summer. There are no RSL locations without active dust devil sightings, except for NE Elysium Planitia (fig. S13). We note that our measurements of dust devil velocity suggest that (i) the daytime wind stresses at RSL locations are systematically underestimated by, for example, the global-scale MCD (Fig. 7 and fig. S13) and (ii) RSL formation—if indeed (partially) driven by atmospheric dynamics—might occur around noon, when dust devil density and near-surface wind velocities are highest.

We note that our measurements can also be used to contextualize dune displacement rates and seasonality, further addressing the martian sand transport puzzle. For example, Roback *et al.* (*96*) report peak dune migration rates for dune fields in two Northern Hemisphere sites in Syrtis Major in southern summer, which is only partially supported by GCM outputs, however (modeled wind velocities remain below ~12 m/s). We observe two dust devils (with azimuth and velocity measurements) in Syrtis Major, both >~26 m/s and >0.03 Pa (both more than 15 m/s faster and with 0.03 Pa higher wind stresses than predicted by the MCD for the background wind field at the same place and time), one in northern summer (*L*_s_ 106°) heading E-SE (agreeing with the MCD prediction by ~±15°) and one in southern summer (255°) heading S (agreeing with the MCD prediction by ~±15°). Our dust devil–derived wind velocities and wind stresses seem to be able to explain why particle saltation and dune migration in the area are generally possible and might occur at different rates in different seasons. Notably, the two observed dust devils are about ~150 km away from the dune fields and might thus not be entirely representative.

### Implications for the continued exploration of Mars

**Fig. 9.**
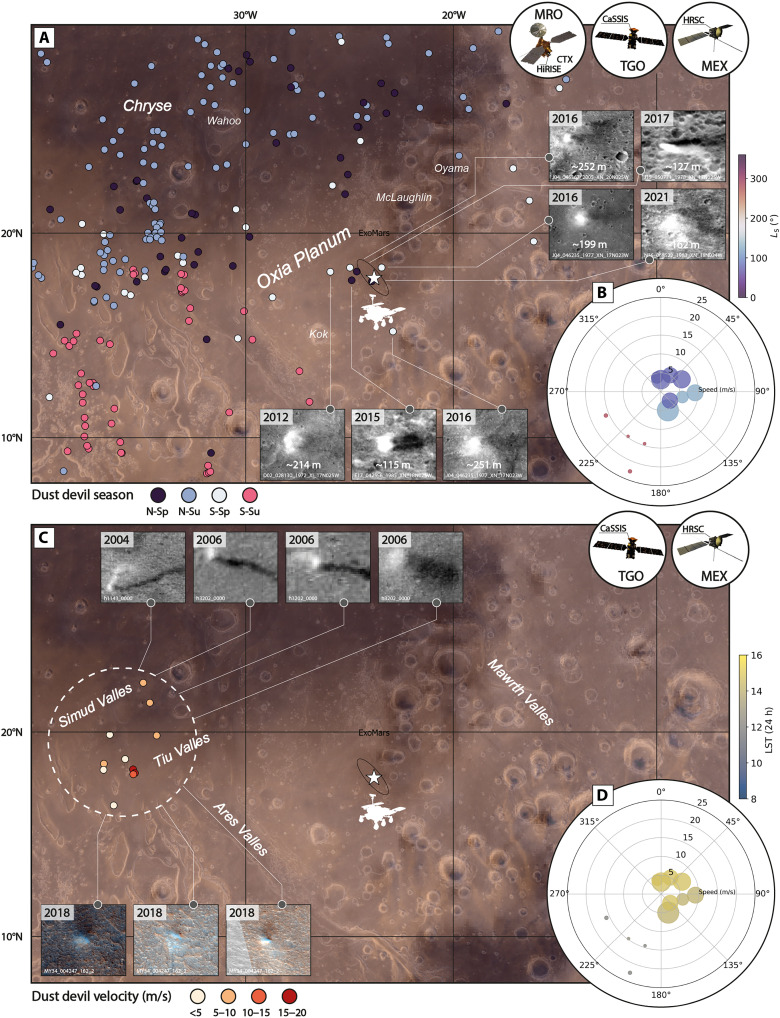
Dust devil occurrence and migration patterns in the wider Oxia Planum area. (**A**) Spatial and seasonal distribution of CaSSIS- HRSC-, and CTX-observed dust devils (*58*) in the wider Oxia Planum area. Viking color mosaic in the background. The year of occurrence of the seven dust devils closest to the ExoMars rover landing ellipse(s) are indicated. The approximate extent of the ExoMars study area (black ellipse) is based on (*117*). (**B** and **D**) CaSSIS- and HRSC-derived polar migration plots; radius indicates migration velocity, theta angle indicates migration azimuth (0° North, 90° East, and so on), and shape size indicates estimated dust devil diameter (small to large). Shape color represents the (B) season or (D) local time of occurrence. (**C**) Velocities of the available dust devils in Tiu and Simud Valles. Note that the migration information was derived from three images only. CTX, HRSC, and CaSSIS images of dust devils are shown in the insets. Image credit: ESA/TGO/CaSSIS CC -BY-SA 3.0 (https://creativecommons.org/licenses/by-sa/3.0/deed.en) IGO, ESA/DLR/FU Berlin CC -BY-SA 3.0 (https://creativecommons.org/licenses/by-sa/3.0/deed.en) IGO; the shown images were cropped from the original CaSSIS and HRSC images.

Our measurements of seasonal and diurnal wind velocity, azimuth, and wind stress have important implications for mission operations and planning efforts, for example, regarding the assessment of the probability of atmospheric solar panel cleaning events and, thus, solar panel efficiency over the lifetime of a landed mission. For instance, the ExoMars rover with its proposed landing site in Oxia Planum (*97*) can expect to experience sporadic dust devil activity, as indicated by the seven close-by, CTX-scale dust devil sightings between 2012 and 2021, i.e., substantially more than InSight with *n* = 0 (*58*) (Fig. 9), as was anticipated by Reiss and Lorenz (*86*). CaSSIS and HRSC did not yet observe active dust devils in the direct vicinity of the ExoMars landing site, but regional observations made in the nearby Tiu and Simud Valles suggest that ExoMars could expect to experience fast-moving (~15 and up to ~23 m/s, ~0.05 Pa) north-easterlies at noon during southern summer and slower-moving (~5 and up to ~9 m/s, ~0.005 Pa) south-westerlies in the early afternoon during northern summer (Fig. 9). Global-scale MCD wind velocity and azimuth predictions generally agree with our measurements, but substantially underestimate the CaSSIS-observed peak velocity at local noon by ~9 m/s.

The combination of vortex activity and wind gusts has contributed to the removal of dust from solar panels and other hardware for missions like Pathfinder/Sojourner, Spirit, Opportunity, Phoenix, Curiosity, and InSight that experienced MCD-predicted (nonvortical) wind velocities between ~7 and ~27 m/s (annual maximum of monthly averages) (*15*, *16*). Our database and that of Conway *et al.* (*58*) suggest that the ExoMars rover should encounter fewer “large” dust devils than Opportunity and Viking 1 and 2, but more than Spirit, Phoenix, Zhurong, Perseverance, Curiosity, and InSight. The presence of dust devils and wind gusts exceeding the assumed wind stress threshold also indicates that ExoMars will be in a unique position to make direct observations of both vortical and nonvortical dust lifting processes, and underlines the importance of planetary protection measures, as terrestrially contaminated dust could be transported over substantial distances from the landing site.

If considering the currently available CaSSIS and HRSC data, the best temporal coverage (i.e., the best compromise between observation period and imaging cadence) is available for Amazonis Planitia, where CaSSIS acquired six subsequent images over the course of 16 Earth days in 2021 (fig. S8). While this level of information is sufficient for the statistical, macroscopic, low-cadence monitoring of regional wind velocity and direction, we note that a more systematic and more frequent monitoring of dust devil occurrence and migration, either by dedicated monitoring campaigns executed by one or several orbiters (e.g., TGO, MEX, and MRO) or by sending dedicated missions, such as enabled by CubeSats with medium-resolution imagers (~6 m pixel scale) and on-board scheduling and data processing (*98*), could produce local and more detailed (and thus reliable) remote sensing–based information about the dynamics of the lowermost atmosphere. The effect of such dedicated orbital monitoring efforts could be substantially amplified by ground-based and airborne monitoring stations that close the temporal gaps left by orbital imaging and that are capable of characterizing the dynamics of the atmosphere (along with other atmospheric parameters) continuously, in specific locations. Integrated and spatiotemporally resolved monitoring—and possibly forecasting—of Mars’ atmospheric dynamics will be vital for future robotic—and specifically human—missions to Mars.

## MATERIALS AND METHODS

### Instruments and datasets

CaSSIS is ESA TGO’s imaging system and acquires color (BLU: 495 nm, PAN: 678 nm, RED: 836 nm, NIR: 939 nm) and stereo images of the martian surface with a pixel scale of about 4.6 m and a nominal footprint width of about 9.5 km (*52*). CaSSIS stereo pairs are acquired using a rotation mechanism that enables along-track, single overflight stereo imaging with a temporal baseline of about 48 s. Here, we use map-projected NPB images (NIR-PAN-BLU, resampled to 4 m/pixel). Since MY 34, CaSSIS has acquired more than 44,000 images (as of February 2024). Not each CaSSIS acquisition offers NIR, PAN, and BLU channels (only about ~89%, *n* = 39,475), but we choose to rely on NPB products (versus single-channel products) because NPB products (i) provide the best signal-to-noise ratio and (ii) color products in general greatly facilitate the detection of (bright, whitish) dust devils due to color fringing artifacts (Fig. 1 and fig. S1). TGO is on a quasi-polar (inclined at 74°), near-circular orbit, which results in a relatively homogeneous distribution of image pixel scales.

HRSC is the main imaging system on ESA’s MEX and is designed for along-track, single overflight, photogrammetric mapping at a pixel scale as small as ~12 m using five panchromatic channels (1× Nadir, 2× Stereo, and 2× Photometry channels, all centered at 675 ± 90 nm) and four color channels [blue centered at 440 nm, green at 530 nm, red at 750 nm, and near-infrared at 970 nm (*53*, *99*)] with a footprint width of about 70 km at an altitude of 350 km, and with a temporal baseline of ~9 to ~19 s between the individual channel acquisitions. MEX is on a highly elliptic orbit, which results in a heterogeneous distribution of footprint width and image pixel scales, ranging from ~12 m to >3000 m. For our analysis, we use all available map-projected (HRSC Level-3) images that have been acquired since MY 27 (2004) with a projected map scale of 12.5 m (76%, i.e., acquired at/near periapsis), 25 m (17%), 50 m (4%), and >50 m (3%), up to orbit 23,950. For the dust devil detection workflow, we exclusively use nadir channel images (“nd3”). For migration measurements, we include Nadir, Stereo, and Photometry channel images (nd, s1, s2, p1, and p2) after photogrammetric adjustment and orthorectification (*99*).

### Deep learning workflow and review

We use a convolutional neural network (CNN)–based single-shot object detection architecture called YOLOv5 (YOLOv5x, You Only Look Once), as openly available at https://github.com/ultralytics/yolov5 (ultralytics) for PyTorch 1.13. YOLOv5 is a well-established and cutting-edge object detection architecture that has been successfully used for a number of global-scale planetary mapping surveys such as concerned with pitted cones, chloride deposits, and rockfalls on Mars (*100*–*103*) and hollows on Mercury (*104*, *105*).

We train two separate CNN detectors, one for CaSSIS color NPB images (72 training labels taken from 25 images, table S2) and one for HRSC single-channel images (84 training labels taken from 15 images, table S3). Each label consists of a rectangular bounding box drawn around a dust devil by a human expert following previously established workflows (*106*–*108*). We include a total of five CaSSIS and five HRSC negatives, i.e., background images without any dust devils to help fine-tune the detectors. The overall scarcity of labels is governed by the limited knowledge of the CaSSIS and HRSC science and operations teams about the presence of dust devils in the overall datasets; owing to the relatively small number of labels, we refer to our approach as a “few shot learning” approach. We follow computer vision best practices and employ ample label augmentation, which includes affine image transformations (i.e., rotation, flipping, shearing, up- and down-scaling ±10%) and radiometric modifications (i.e., brightness, contrast, and hue modifications). We train both detectors using an NVIDIA RTX 3090 graphical processing unit (GPU); detector training takes about ~10 min.

We evaluate the performance of the trained detectors using labeled testing data (not included in the training), 10 HRSC labels (~10% of overall dataset) taken from five images (including three negative images), and 7 CaSSIS labels (~10% of overall dataset) taken from five images (also including three negative images, fig. S14). Both detectors achieve excellent performances in the test set: a recall (% dust devils detected in the test set) of ~70% and ~80%, and a precision (% of detections correct) of ~80% and ~100%, both at a confidence score of 0.6 (with a default Intersection-over-Union of 0.5), and an average precision (integrated relation of recall and precision over the full range of confidence scores) of 84 and 98% (fig. S14). The confidence score describes the confidence of the detector in its detection (posterior probability): A high score indicates a detection with a higher probability of being a correct detection (true positive), and a low score indicates a detection with a lower probability of being a correct detection. Usually, cutting off detections at higher confidence scores increases the overall precision of the results (less false positives) but to the cost of an overall reduced recall (fewer detections overall); in contrast, cutting off detections at a lower confidence score increases the recall (more detections overall), but to the cost of a reduced precision (more false positives). Here, we identify a confidence score of 0.6 as the optimal compromise between recall and precision, which maximizes the number of dust devil candidates while limiting the probability for false positives to occur. A moderate amount of false positives greatly facilitates the expert review of the CNN outputs, as detailed below. Here, the term “candidate” describes dust devil detections that have not been verified by a human expert yet and, thus, feature a certain probability of being incorrect.

We deploy both detectors in a preexisting data processing pipeline (*109*–*111*) and process a total of 39,475 CaSSIS (up to orbit 27,816, February 2024) and 5390 HRSC images (up to orbit 23,950, January 2023) in ~10 days using one single NVIDIA RTX 3090 GPU. The outputs of the pipeline include a sequence of meta-information for each detected dust devil candidate (e.g., longitude, latitude, image *L*_s_, image LST, and image Coordinated Universal Time (UTC) as well as (full-resolution) .png or .tif thumbnails representing crop-outs of the respective dust devils from their parent images along the CNN-predicted bounding box (see Fig. 1 and fig. S14). Following the workflow developed and used by previous work (*12*, *58*, *100*,  *102*, *103*, *111*), the thumbnails of all of the CNN-derived candidate dust devils are manually examined by a human expert, candidate by candidate; false detections are removed from the final dataset and are not included in the scientific analysis.

The CNN-predicted bounding box around each identified dust devil (i.e., the dimensions of the thumbnails) is loosely related to the diameter of the respective dust devil vortex: Generally, small dust devils feature small bounding boxes, and large dust devils feature large bounding boxes (fig. S14). To improve the accuracy of that relation, we manually measure the physical diameter of a random sample of 100 CaSSIS and HRSC dust devils and relate them to the CNN-derived bounding box diameters (diagonals), converted from pixel space to a physical length using the spatial resolution of the respective image (fig. S15). We follow the dust devil diameter measurement approach as established by Conway *et al.* (*58*) and measure the physical size of the diameter of the dust devil column where the vortex is liberated as a dust cloud. The relation between dust devil diameter and bounding box diameter can be described by a linear fit [as established by Bickel *et al.* (*106*, *107*)]; we use these relations to correct all CNN-derived dust devil diameter estimates (fig. S15). We note that the stark contrast of CaSSIS and HRSC data pixel scales (4 versus >12.5 m) affects what size of dust devil the instruments are sensitive to and are likely to be reflected in the produced estimated dust devil diameter populations.

### Migration measurements and validation

The dynamic movement of dust devils can cause artifacts in the CaSSIS and HRSC image data. In CaSSIS and HRSC stereo images, the part of the surface that is covered by moving dust devils usually cannot be used for the stereo correlation (i.e., local decorrelation), which leads to small gaps in the resulting topographic data. In CaSSIS color images, the movement of dust devils can cause a local misalignment of the color channels and thus color fringing (fig. S1). Here, we use those artifacts to measure dust devil displacement directions (referred to as “azimuth”) and speeds (referred to as velocity).

Generally, we measure azimuth and velocity by relating one or more measures of the change(s) of dust devil position (coordinates in image 1 and 2 or *n*) to the time that passed between the individual map-projected (and accurately aligned) acquisitions. Here, the recorded position of a dust devil is the point where the core of the vortex touches the surface, as marked by the shadow cast by the vortex itself. Depending on what specific data are available for a given detected dust devil, we use two different approaches: (i) for CaSSIS and HRSC stereo images, we measure the position of the dust devil in stereo images 1 and 2 (two position measurements, one time difference on the order of ~48 s, CaSSIS) or in stereo images s1, p1, nd, p2, and s2 (five position measurements, four time differences on the order of ~13 s each, HRSC); (ii) for CaSSIS color fringe, i.e., where no stereo pair is available, we measure the position of the dust devil in the NIR and PAN channels (two position measurements, one time difference on the order of ~1 s) (fig. S16).

Notably, CaSSIS color fringe measurements are easier to make for fast dust devils, as they experience larger spatial displacements and thus more pronounced color fringes; for slow dust devils, color fringe measurements might not be possible at all. Similarly, the quality of the various HRSC stereo channels (s1, p1, nd, p2, and s2) does not always allow for accurate measurements. Whenever the data are not appropriate for accurate measurements, we refrain from extracting azimuth and velocity information. We provide the velocity measurements in meters per second and the azimuth measurements in degrees, with 0° and 360° pointing toward the north, 90° toward the east, and so on. We compute the acceleration of individual dust devils using the three velocity changes measured by HRSC over the four individual measurement intervals: s1-p1 to p1-nd, p1-nd to nd-p2, and nd-p2 to p2-s2.

Owing to the accurate alignment of our map-projected products, the accuracy of our displacement measurements is only affected by the accuracy of our manual dust devil displacement measurements. We quantify this accuracy to be on the order of 1 pixel for color fringe measurements and 3 pixels for stereo measurements, i.e., 4 m for the CaSSIS color fringe method, 12 m for the CaSSIS stereo method, and between 37.5 and 150 m for the HRSC stereo method. With this accuracy, we determine the error of our velocity measurements as ±~4 m/s for CaSSIS color fringe measurements, ±~0.2 m/s for CaSSIS stereo measurements, and ±~1.3 m/s for HRSC stereo measurements.

We cross-validate the velocity and azimuth measurements derived by CaSSIS stereo and color fringe approaches using all dust devils that feature both stereo and color (NIR and PAN) coverage known to the CaSSIS science team as of January 2025 (i.e., 35 dust devils in 14 CaSSIS stereo pairs). On average, the results derived by both approaches agree very well, with a median azimuth difference of 13.0° and a median velocity difference of 2.2 m/s (fig. S17). Our results indicate that the CaSSIS color fringe method might slightly overestimate the velocity of slow dust devils (<~10 m/s), but might slightly underestimate the velocity of fast dust devils (>~10 m/s). We note that dust devils might change their velocity between the stereo 1 and stereo 2 acquisitions, leading to accurate yet slightly different measurements of velocity and azimuth derived from stereo 1–stereo 2 versus stereo 1 NIR–stereo 1 PAN (or stereo 2 NIR–stereo 2 PAN).

We are not able to directly compare CaSSIS and HRSC measurements due to a lack of available images covering the exact same dust devils. The CaSSIS and HRSC science and operations teams are currently attempting the acquisition of images with overlap in space and (absolute) time to enable a direct cross-comparison of CaSSIS and HRSC measurements. As a preliminary proxy, we compare azimuth and velocity measurements derived with all three methods from one CaSSIS and two HRSC images acquired over three consecutive days over the same location in the Erebus Montes region in December 2024 (*L*_s_ 12°, LST ~2 p.m. for all images, fig. S3); these images and measurements represent the (current) closest spatiotemporal match of CaSSIS and HRSC: On December 7, HRSC measures an average azimuth of 134° and a velocity of 6.3 m/s; on December 8, CaSSIS measures an azimuth of 28.5 and 15° as well as a velocity of 1.5 and 1.6 m/s (stereo and color fringe, respectively); and on December 9, HRSC measures an azimuth of 244° and a velocity of 6.6 m/s. Apparently, the wind field in the Erebus Montes region experienced a substantial, dynamic shift, with a rotation of the wind field from NE, over N-NE, to W-NW; in parallel, the wind field decelerated, then reaccelerated. Besides representing a relevant observation of the dynamics of the lowermost martian atmosphere, the dynamic shift of the wind field affects the reliability of the CaSSIS-HRSC cross-comparison, highlighting the demand for simultaneous observations of the exact same dust devil population by both CaSSIS and HRSC (fig. S3).

As an alternative, statistical proxy, we compare all azimuth and velocity measurements as derived using CaSSIS stereo and HRSC stereo approaches for the global dust devil dataset (fig. S18). Both populations agree exceedingly well, with nearly identical median values (velocity, CaSSIS: 8.4 m/s; HRSC: 8.9 m/s), very similar maximum values (velocity, CaSSIS: 39.1 m/s; HRSC: 42.7 m/s), and identical azimuth ranges (both ~0 to 360°, fig. S18). HRSC resolves larger dust devils, on average, which is likely driven by the substantially larger footprint on the surface and lower spatial resolution; We note that this does not affect our migration measurements in any way.

We also compare our HRSC stereo migration velocity and dust devil diameter measurements with other, independent HRSC measurements performed by earlier, regional surveys (*60*). Specifically, we compare measurements for images h2101_0000 (Stanzel mean: ~16 m/s and ~270 m, our mean: ~21 m/s and ~130 m), h2133_0000 (Stanzel mean: ~13 m/s and ~230 m, our mean: ~10 m/s and ~148 m), h3202_0000 (Stanzel mean: ~4 m/s and ~269 m, our mean: ~4 m/s and ~119 m), and h3246_0000 (Stanzel mean: ~7 m/s and ~173 m, our mean: ~6 m/s and ~110 m). Both populations of velocity measurements agree very well, but Stanzel *et al.*’s (*60*) dust devil diameter estimates are a factor of ~1.9 larger than our estimates, on average. Stanzel *et al.*’s (*60*) measurements were predominantly conducted in 25 m/pixel images; for one dust devil that was also imaged with 5 m/pixel (HRSC SRC, “Super Resolution Channel”) they report a factor of ~2.2 overestimation of that dust devil’s diameter measurement in the lower-resolution (25 m/pixel) data. On the basis of Stanzel *et al.*’s (*60*) observation and the fact that 98% of our dust devil migration measurements were made in higher-resolved data (12.5 m/pixel), we conclude that our diameter estimates are likely more accurate. Notably, our dust devil detectors identified a majority of Stanzel *et al.*’s (*60*) dust devils, but we did not consider a substantial portion of the image data reliable enough for migration measurements, such as for HRSC images h2242_0000 and h3210_0000, which is why those data are not included in our analysis. The photogrammetric adjustment techniques we apply to analyze and geometrically correct HRSC images (*99*) were not available yet for the work of Stanzel *et al.* (*60*).

Last, we compare and validate our orbital migration data with in situ data recorded by the Viking 1 lander (Fig. 4). Both datasets agree exceedingly well; the CaSSIS and HRSC data capture both the evolution of the wind speed and the rotation of the wind speed azimuth from a NE, over N, to SW direction over the course of the day. We note that Viking 1 is the only lander that features both rich meteorological data and CaSSIS and HRSC dust devil detections (Fig. 1), representing the only opportunity for a direct, meaningful comparison of in situ and orbital data.

### Conversion to wind shear velocities and wind stress

We convert all measured lateral dust devil velocities to lateral shear velocities (or wind friction velocities) assuming that dust devil velocity represents the ambient wind velocity at a height of 10 m above surface level, following (*27*, *60*, *65*, *67*, *87*, *92*). We identify several examples of dust devils that move at the same speed as directly colocated, diffuse, (nonvortical) near-surface clouds, further supporting this assumption (fig. S19). Wind velocity ( $\bar{U_{x}}$ ) at height *z* can be converted to wind shear velocity ( $u_{*}$ , at surface level)—and vice versa—using the “law of the wall” along with several key assumptions

$$\bar{U_{x}}\;\left(z\right)=\frac{u_{*}}{\kappa }\text{ln}\left(\frac{z}{z_{0}}\right)$$ (1)

with κ as the von Kármán constant (0.40) and $z_{0}$ as the aerodynamic surface roughness (*36*). The law of the wall is the most widely used equation to relate wind velocity to shear velocity (*30*, *36*, *49*) and is generally considered to be sufficient for studies related to near-surface saltation (*112*). It best applies to flat, homogeneous surfaces and assumes that wind velocity measurements are taken above the saltation layer, which is generally true for this particular study. We note that Eq. 1 requires a convectively stable atmosphere, which is not entirely accurate in the presence of dust devil vortices, which will affect the overall accuracy of the outputs of Eq. 1. We follow (*49*) and (*113*) in computing$\;z_{0}$ with

$$z_{0}=2*\frac{D}{30}$$ (2)

using *D* = 30 mm as a value that represents the overall martian median grain size, reflecting a compromise between Sullivan *et al.* (*30*) and Charalambous *et al.* (*49*), who studied very fine- and very coarse-grained landing sites (InSight versus Spirit), which might be most representative for Mars as a whole. We note that utilization of one single $z_{0}$ value is an oversimplification that is driven by the lack of knowledge about the actual grain size distribution at the identified dust devil locations, which will also affect the overall accuracy of the outputs of Eq. 1.

We use MCD predictions for the atmospheric density $\rho_{a}$ at an altitude of 10 m above the surface and the derived wind shear velocity to estimate the wind stress that is acting on the surface using (*29*, *36*)

$$\tau =\rho_{a}*u_{*}^{2}$$ (3)

The relation between $\bar{U_{x}}$ and $u_{*}$ is dominated by the stability of the atmosphere and the vertical wind profile, which makes wind stress—that considers atmospheric density—the most physical and representative quantity that can be thresholded to identify dust lifting conditions on a global scale (*29*). We underline that Eqs. 1 to 3 rely on a series of assumptions and necessary simplifications that will affect the overall accuracy of the derived wind stress values.

### Estimation of dust lifting rates

For our estimates of dust lifting rates, we deploy a power law relation between estimated dust devil size (*D*) and lifetime (*l*) as well as data put forward by Reiss *et al.* (*66*) and based on Greeley *et al.* (*44*) and Stanzel *et al.* (*60*)

$$l=28.13\;s/m*D^{0.62}$$ (4)

A similar power law relation has been identified by Lorenz (*114*) using field and orbital measurements on Earth and Mars. In addition, we use a linear relation between dust devil lifetime (*l*) and travel distance (*L*), as established by the data presented by Stanzel *et al.* (*60*)

L=6.075m/s∗l+462 (5)

Those relations result in mean lifetimes of 7 min and mean travel distances of 3000 m for the CaSSIS and HRSC dataset.

Following Stanzel *et al.* (*60*), we combine our estimates of lifetime and travel distance (per dust devil, using Eqs. 3 and 4) with our estimates of dust devil diameter (*D*) to compute the area of the surface modified by the dust devil $A_{\text{mod}}$

$$A_{\text{mod}}=D*L$$ (6)

resulting in a mean modified surface area of 0.28 km^2^ for the CaSSIS and HRSC dataset.

We use the estimated dust devil lifetime, modified surface area, two dust fluxes derived from lander data [2 × 10^−5^ and 5 × 10^−4^ kg m^−2^ s^−1^, per Greeley *et al.* (*44*) and Metzger *et al.* (*83*)], and two hemispherical estimates of dust devil density [0.06 and 0.6 dust devils km^−2^ MY^−1^ in the north and south, respectively, per Whelley and Greeley (*85*)] to estimate the dust lifting rate of our dataset, reproducing the workflow outlined by Stanzel *et al.* (*60*).

### MCD data extraction

The MCD (*55*, *115*) uses GCMs to model and predict climatological variables on regional scales, including wind velocity and azimuth, with a spatial sampling of 5.625° in longitude and 3.75° in latitude. The MCD uses a “high resolution topography mode” to refine its predictions of surface and vertical pressure, along with a range of other atmospheric variables (excluding wind velocity and azimuth). Its global-scale implies that MCD predictions of the ambient (nonvortical) wind azimuth and velocity represent averages over large regions. In addition, MCD predictions are expected to be less representative of the lowermost atmosphere (*55*). We use MCD version 6.1 (https://www-mars.lmd.jussieu.fr/mcd_python/) and extract values at an altitude of 10 m above the surface, following (*60*, *65*, *67*) using the specific UTC times a given dust devil was observed (translated to a specific LST and *L*_s_) in combination with the associated Dust/EUV (Extreme UltraViolet) scenarios for the corresponding MY.

## Supplementary Materials

### The PDF file includes:

Figs. S1 to S19 Tables S1 to S3 Legend for data S1 Legends for animations S1 to S3

### Other Supplementary Material for this manuscript includes the following:

Animations S1 to S3
