# Supplementary material for: Dust devil migration patterns reveal strong near-surface winds across Mars
Source: Sci Adv. 2025 Oct 8;11(41):eadw5170. doi: 10.1126/sciadv.adw5170 (PMC12506970; doi:10.1126/sciadv.adw5170)
Supplement: Supplementary file 1 — Figs. S1 to S19 Tables S1 to S3 Legend for data S1 Legends for animations S1 to S3 [file sciadv.adw5170_sm.pdf]

Science Advances
AAAS

Supplementary Materials for

Dust devil migration patterns reveal strong near-surface winds across Mars

Valentin T. Bickel *et al.*

Corresponding author: Valentin T. Bickel, valentin.bickel@unibe.ch

Sci. Adv. **11**, eadw5170 (2025)
DOI: 10.1126/sciadv.adw5170

The PDF file includes:

Figs. S1 to S19
Tables S1 to S3
Legend for data S1
Legends for animations S1 to S3

Other Supplementary Material for this manuscript includes the following:

Animations S1 to S3

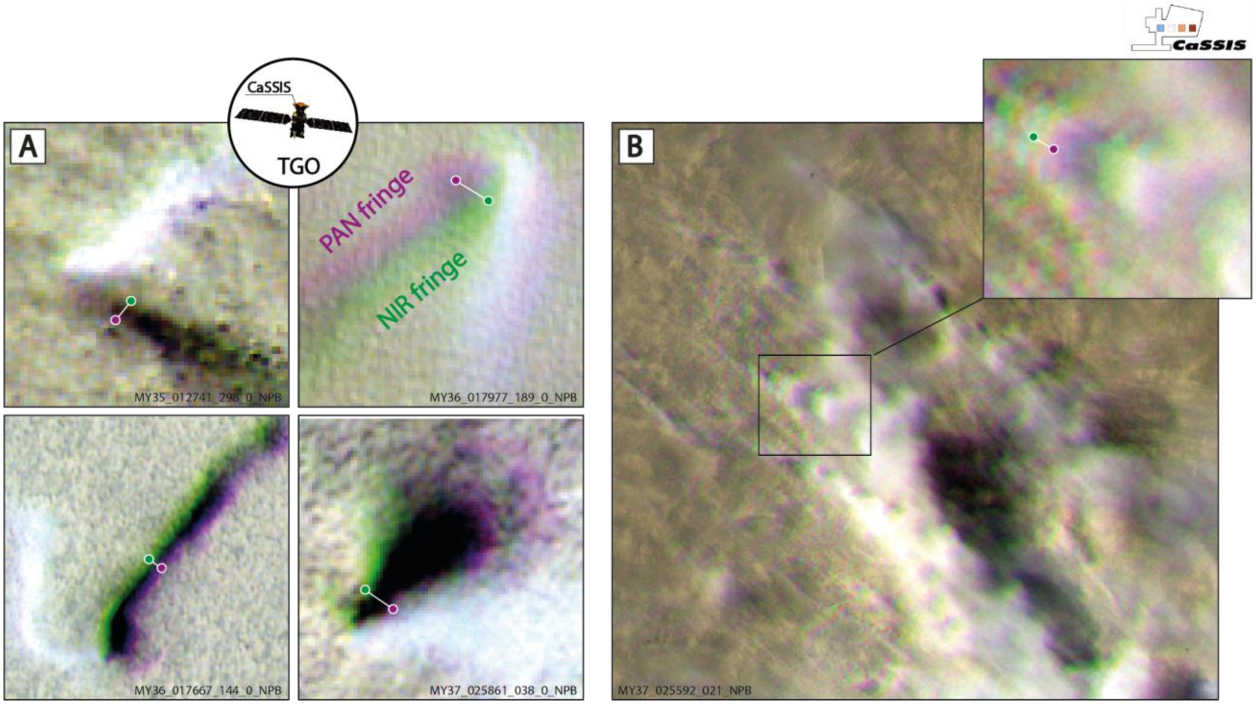

Fig. S1. Examples of CaSSIS color fringing artefacts. (**A & B**) Examples of CaSSIS color NPB (NIR, PAN, BLU channels) images that show color fringing artefacts, for dust devils and dust clouds. Spacecraft badges mark data derived by different missions/instruments. Image credit: ESA/TGO/CaSSIS CC-BY-SA 3.0 IGO; the shown images were cropped from the original CaSSIS images.

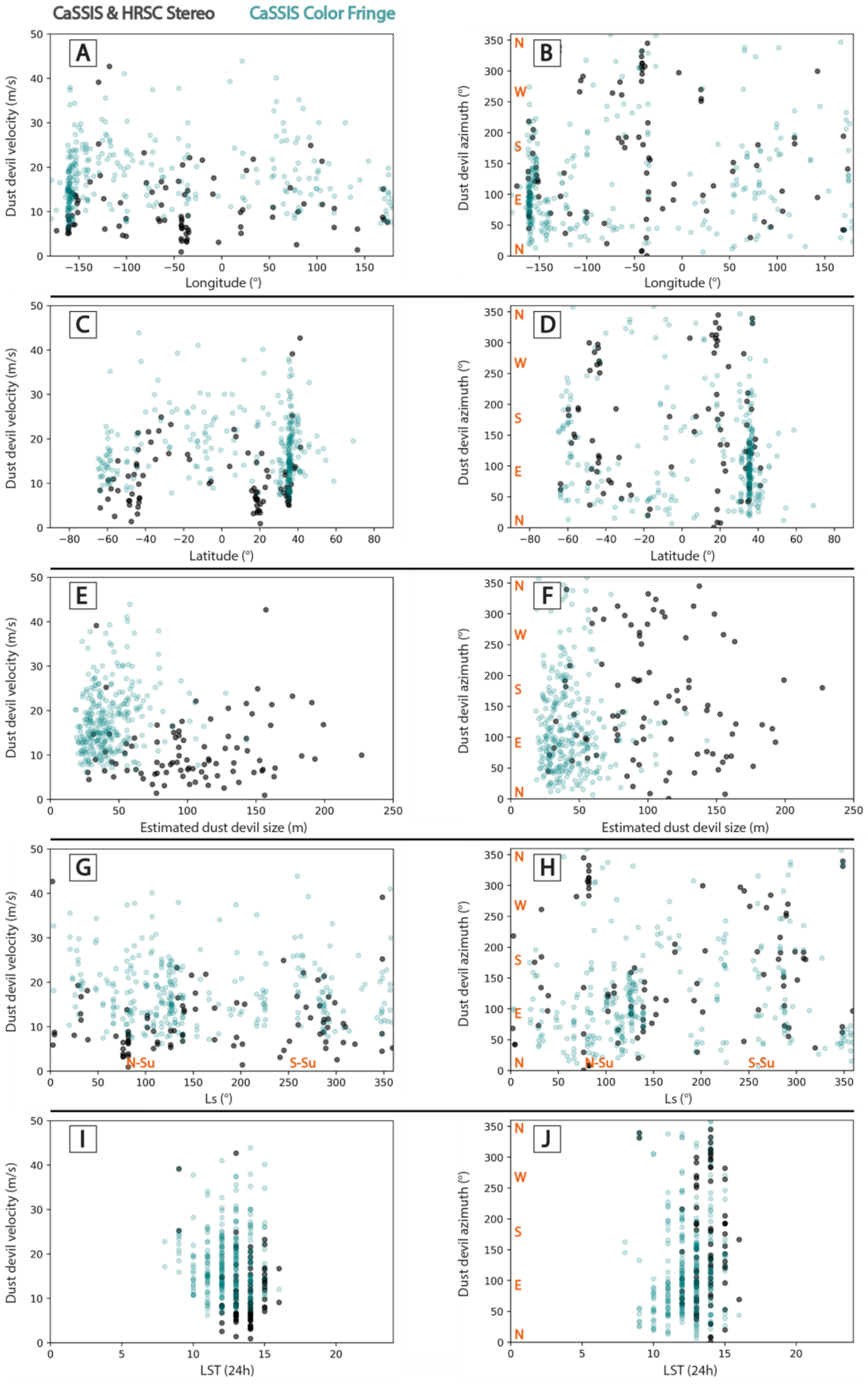

Fig. S2. Correlation of selected dust devil dataset properties. Correlation of CaSSIS & HRSC stereo (black) and CaSSIS fringe (teal) dust devil velocity (left) and azimuth (right) with (**A-B**) longitude, (**C-D**) latitude, (**E-F**) estimated dust devil size (4 'outliers' >250 m are omitted for graphical reasons), (**G-H**) Ls, and (**I-J**) LST.

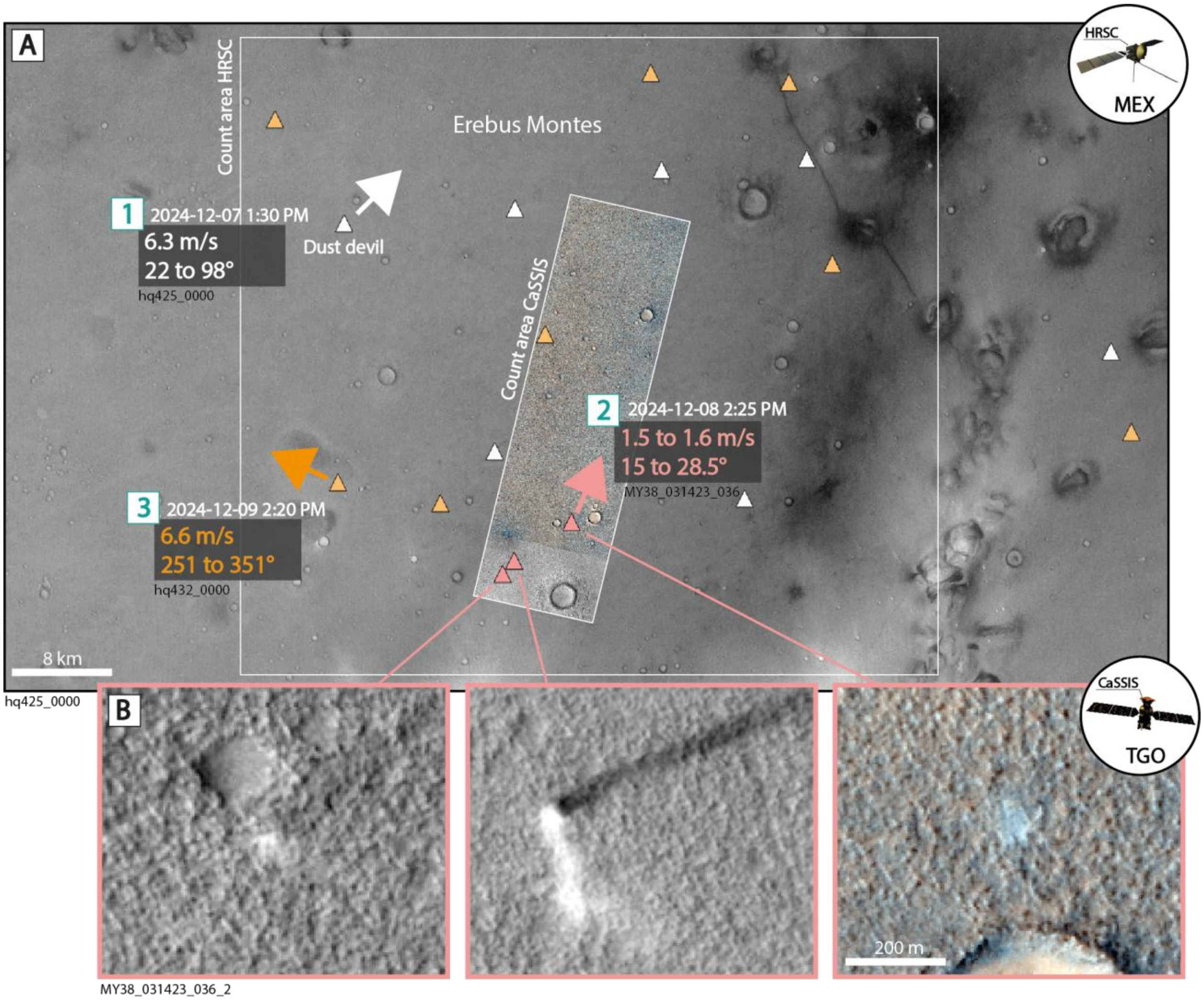

Fig. S3. Coordinated monitoring of day-to-day dust devil abundance and migration behavior. (A) HRSC image hq425_0000 taken on December 7th, locations of resolved dust devils indicated by colored triangles (white: HRSC 2024-12-07, red: CaSSIS 2024-12-08, orange: HRSC 2024-12-09); average migration direction and velocity indicated by arrows and info boxes. Count areas used for dust devil density calculations indicated by white rectangles. HRSC image hq432_0000 not shown for graphical reasons. (**B)** zoomed-in images of the three dust devils resolved by CaSSIS on December 8th; note that only one dust devil was resolved in full color. North is up in all images. Image credit: ESA/TGO/CaSSIS CC-BY-SA 3.0 IGO, ESA/DLR/FU Berlin CC-BY-SA 3.0 IGO; the shown images were cropped from the original CaSSIS and HRSC images.

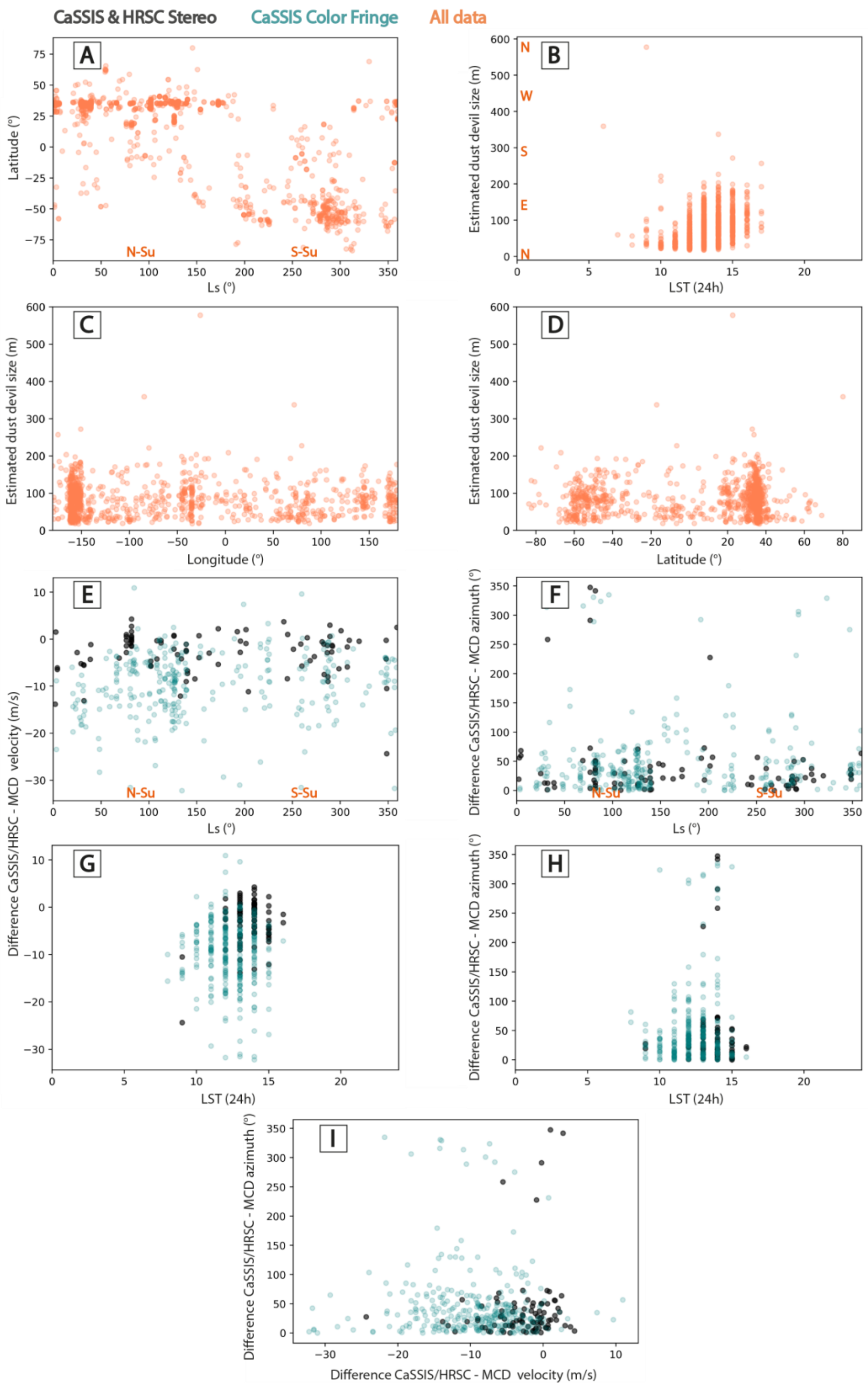

Fig. S4. Correlation of selected dust devil dataset properties. (**A-D**) Correlation of all CaSSIS & HRSC dust devils (orange): latitude, longitude, Ls, estimated size, LST. (**E-I**) Correlation of CaSSIS & HRSC stereo (black) and CaSSIS fringe (teal) dust devils: Ls, LST, difference CaSSIS/HRSC-derived and MCD-predicted speed/azimuth, difference CaSSIS/HRSC-derived speed and azimuth.

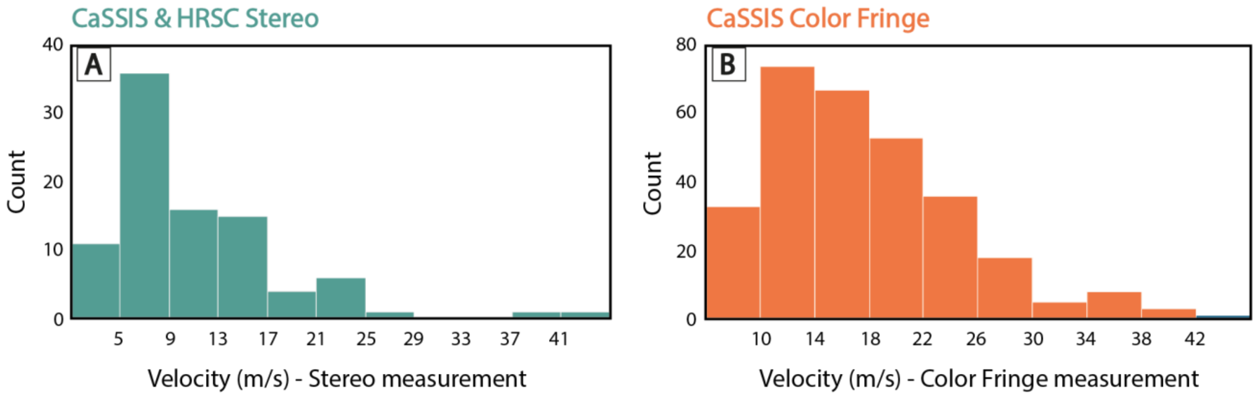

Fig. S5. Distribution of CaSSIS- and HRSC-observed dust devil velocities. Velocities are reported for CaSSIS and HRSC stereo measurements (teal) (**A**) and CaSSIS color fringe measurements (orange) (**B**).

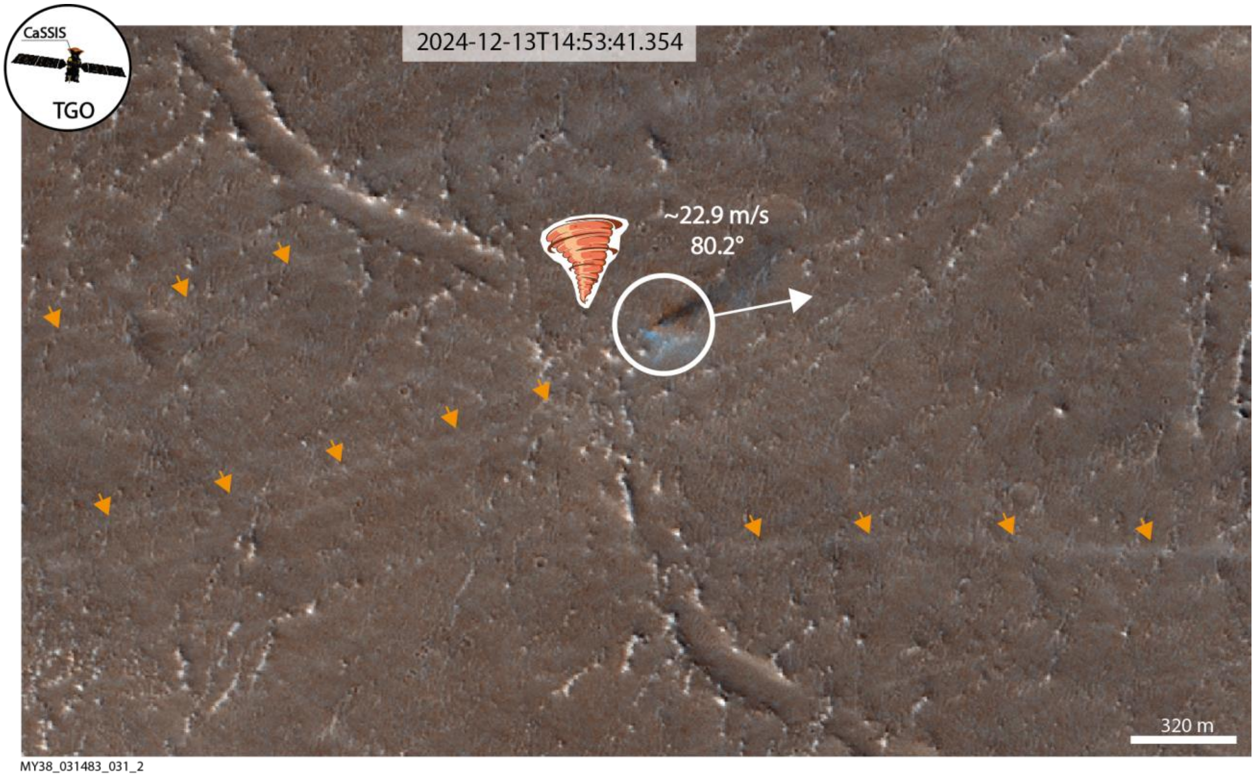

Fig. S6. Observation of dust devil migration azimuth and track orientation. CaSSIS image showing the northeast-ward migration of one dust devil (white circle) that closely agrees with the orientation of the tracks left by previous dust devils (bright tracks). Image credit: ESA/TGO/CaSSIS CC-BY-SA 3.0 IGO; the shown image was cropped from the original CaSSIS image.

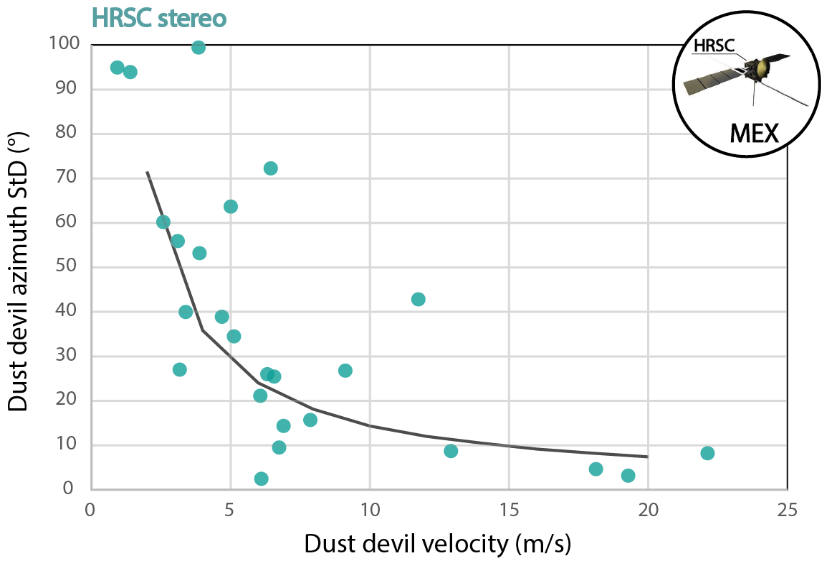

Fig. S7. Dust devil migration azimuth variation as a function of velocity. HRSC-derived dust devil velocities are related to their respective azimuth StD (teal dots). Superposed is an arctangent model (black line) that is based on terrestrial dust devil observations [75].

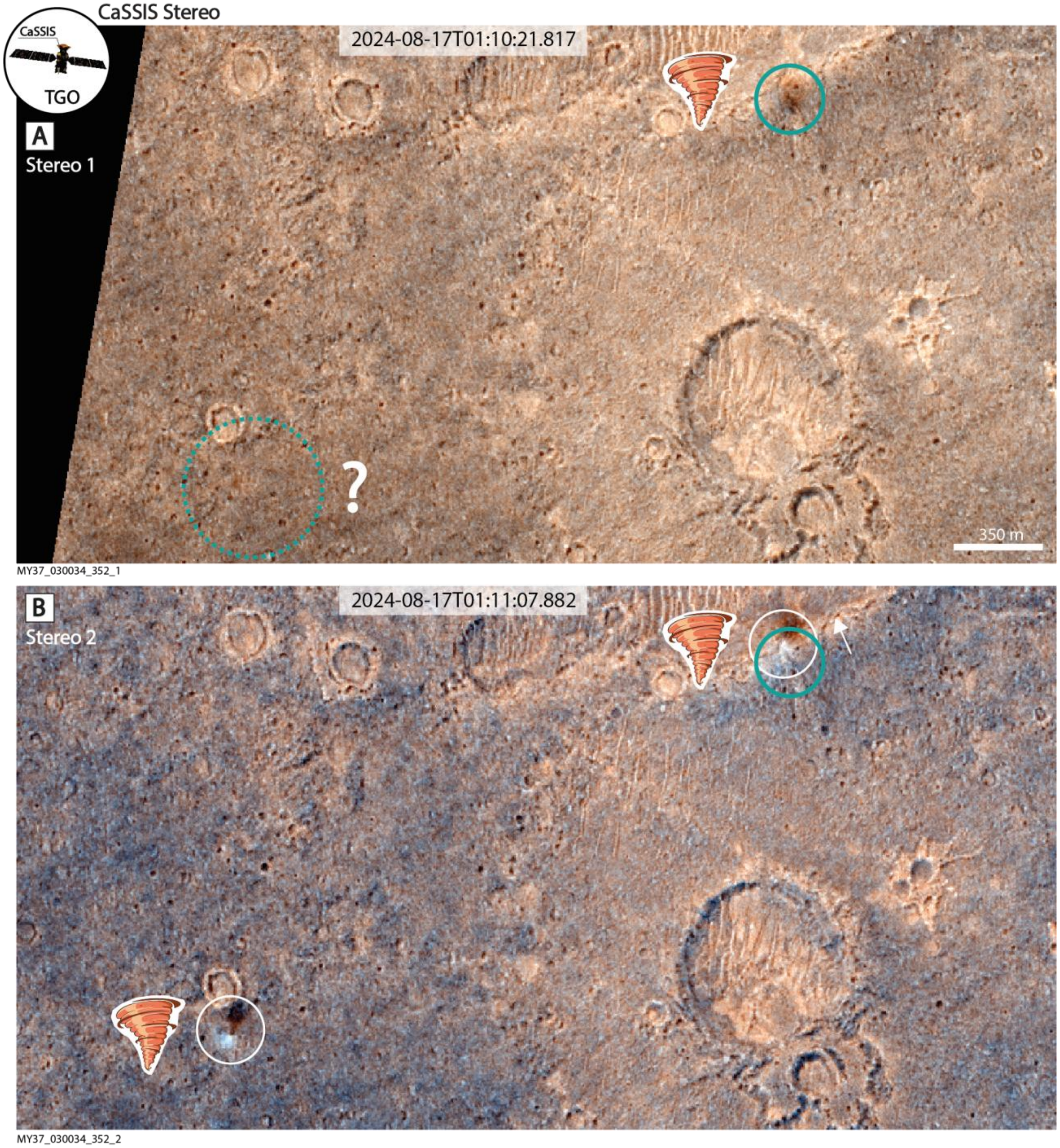

Fig. S8. Potential observation of sporadic dust devil formation. CaSSIS stereo pair showing the slow, northward migration of one dust devil (teal-white circle) and potential formation of a second dust devil, with a temporal difference of ~46 seconds. Image credit: ESA/TGO/CaSSIS CC-BY-SA 3.0 IGO; the shown images were cropped from the original CaSSIS images.

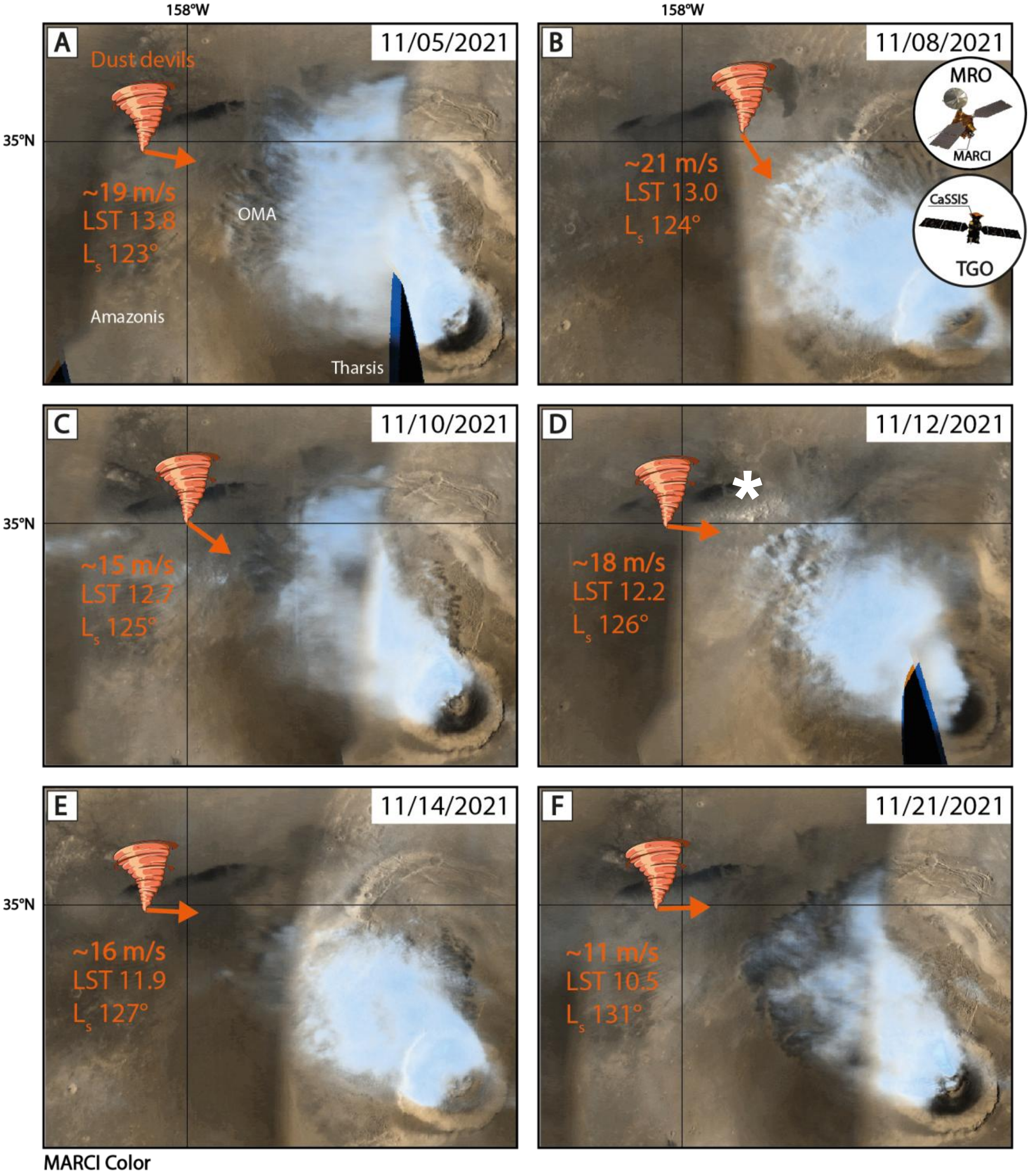

Fig. S9. Month-long sequence of dust devils co-located with a dust lifting event/haze in Amazonis. (**A-F**) A month-long sequence of several fast dust devils (orange shapes, LST, L_s, velocity, and azimuth indicated) detected in six subsequent CaSSIS images and a MARCI-observed dust lifting event/haze in 2021 (white asterisk, particularly pronounced in panel D, north-west of the OMA), plotted on MARCI color composites acquired at the same day of occurrence. Arrow indicates dust devil azimuth (dust devil is located at the tip of the orange vortex). Note the abundance of large, bright H_2O/CO_2 clouds that are not related to the dust lifting event. MARCI image width is ~2500 km. MARCI data gaps are black. Image credit: NASA/MSSS/Arizona State University.

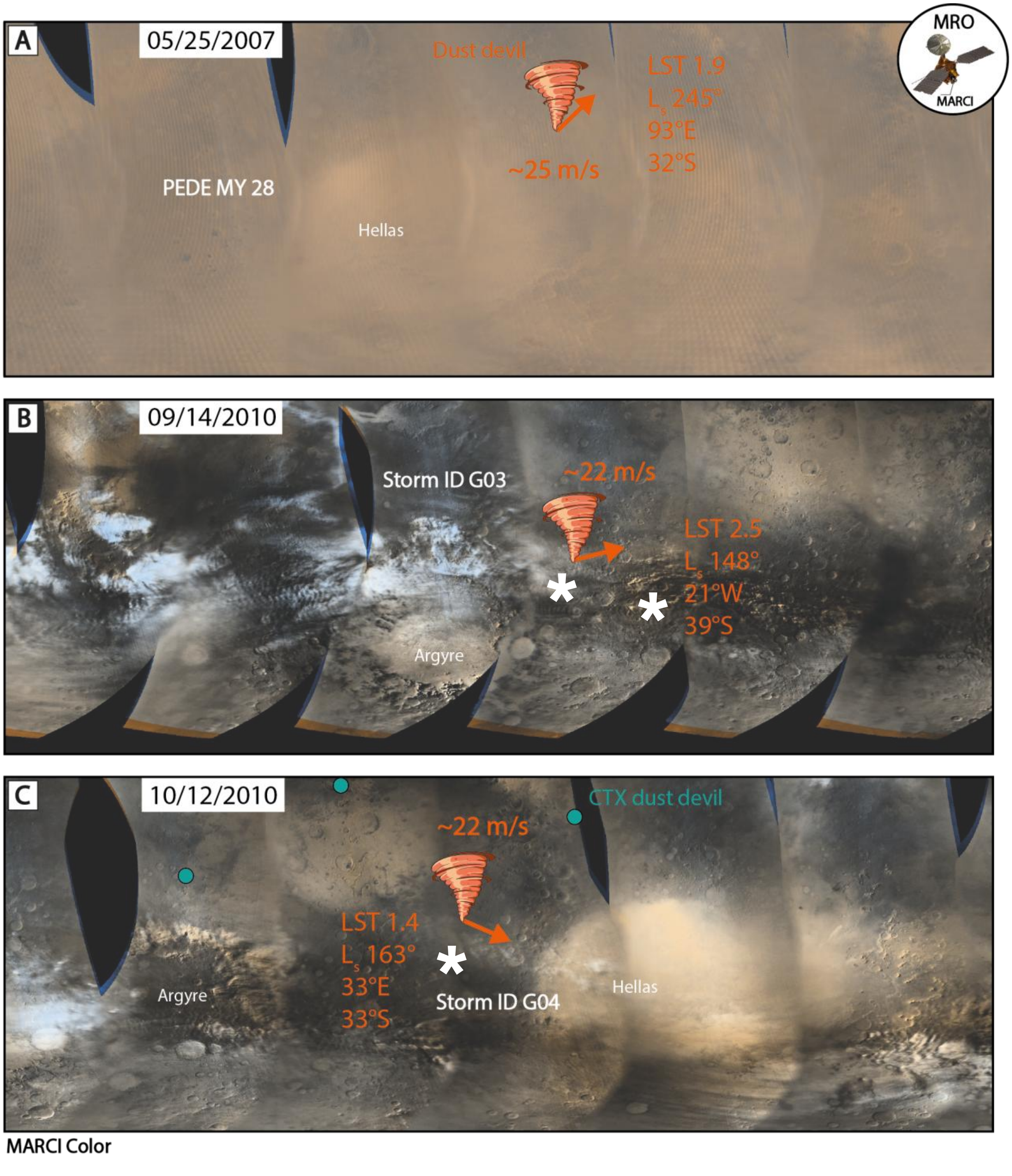

Fig. S10. Spatiotemporal co-location of fast dust devils and dust storms/clouds or dust lifting events/haze. (**A-C**) Spatiotemporal co-location (orange shapes, location, LST, L_s, velocity, and azimuth indicated) of fast CaSSIS & HRSC dust devils and MARCI-observed dust storms/clouds or dust lifting events/haze in 2007 and 2010 (white asterisks), plotted on MARCI color composites acquired at the same day of occurrence. (**A**) shows a planet-encircling dust event (PEDE); (**B-C**) show dust storms (ID G03 and G04) identified by [82]. Arrow indicates dust devil azimuth (dust devil is located at the tip of the orange vortex). Note the abundance of large, bright H_2O/CO_2 clouds that are not related to the dust lifting event. MARCI data gaps are black. Dust devils detected by CTX in the same month (no migration information) are indicated by teal shapes [58]. MARCI image width is ~10,000 km. Image credit: NASA/MSSS/Arizona State University.

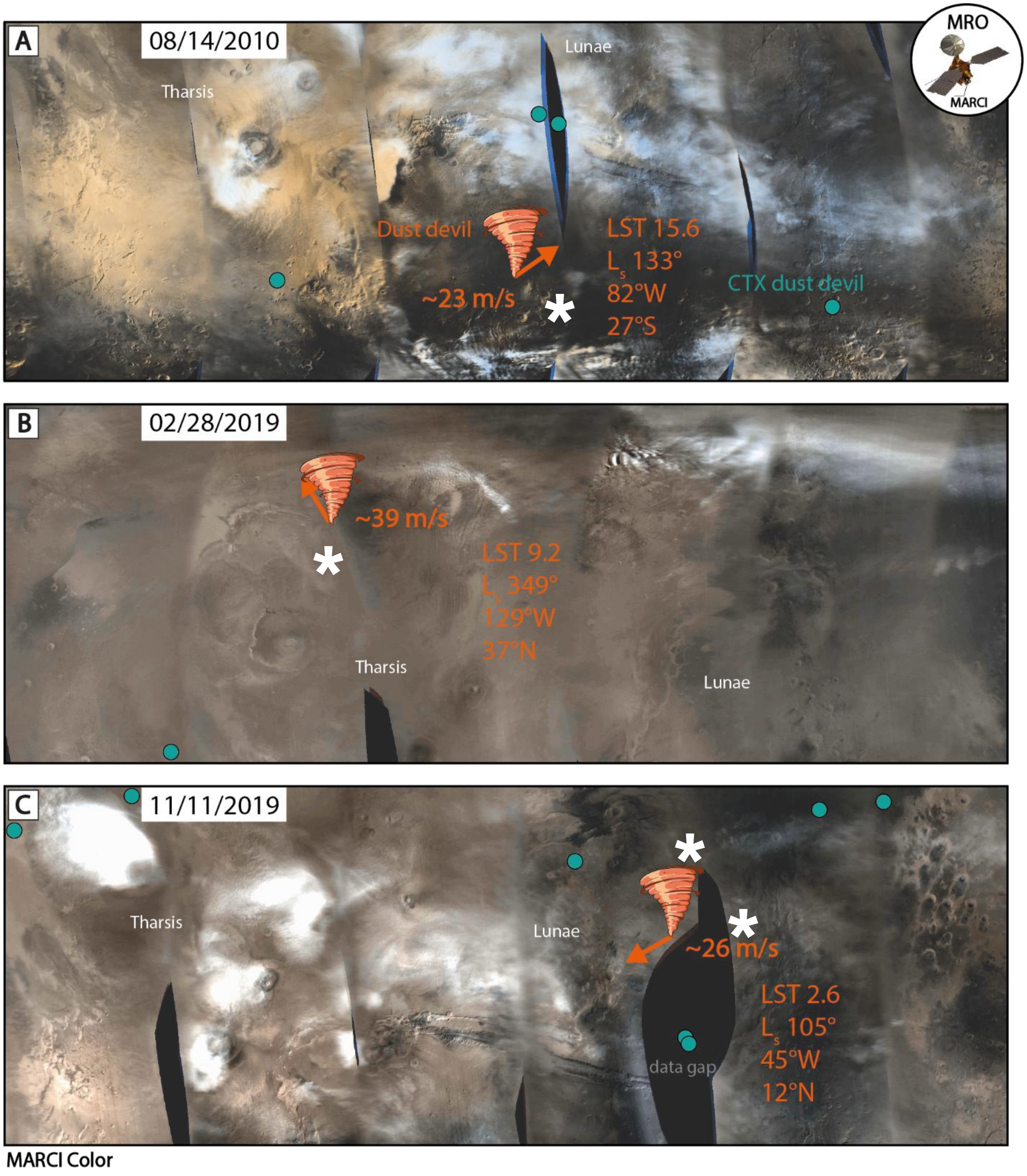

Fig. S11. Potential spatiotemporal co-location of fast dust devils and dust storms/clouds or dust lifting events/haze. (**A-C**) Potential spatiotemporal co-location (orange shapes, location, LST, L_s, velocity, and azimuth indicated) of fast CaSSIS & HRSC dust devils and MARCI-observed dust storms/clouds or dust lifting events/haze in 2010 and 2019 (white asterisks), plotted on MARCI color composites acquired at the same day of occurrence. Arrow indicates dust devil azimuth (dust devil is located at the tip of the orange vortex). Note the abundance of large, bright H_2O/CO_2 clouds that are not related to the dust lifting event. MARCI data gaps are black. Dust devils detected by CTX in the same month (no migration information) are indicated by teal shapes [58]. MARCI image width is ~10,000 km. Image credit: NASA/MSSS/Arizona State University.

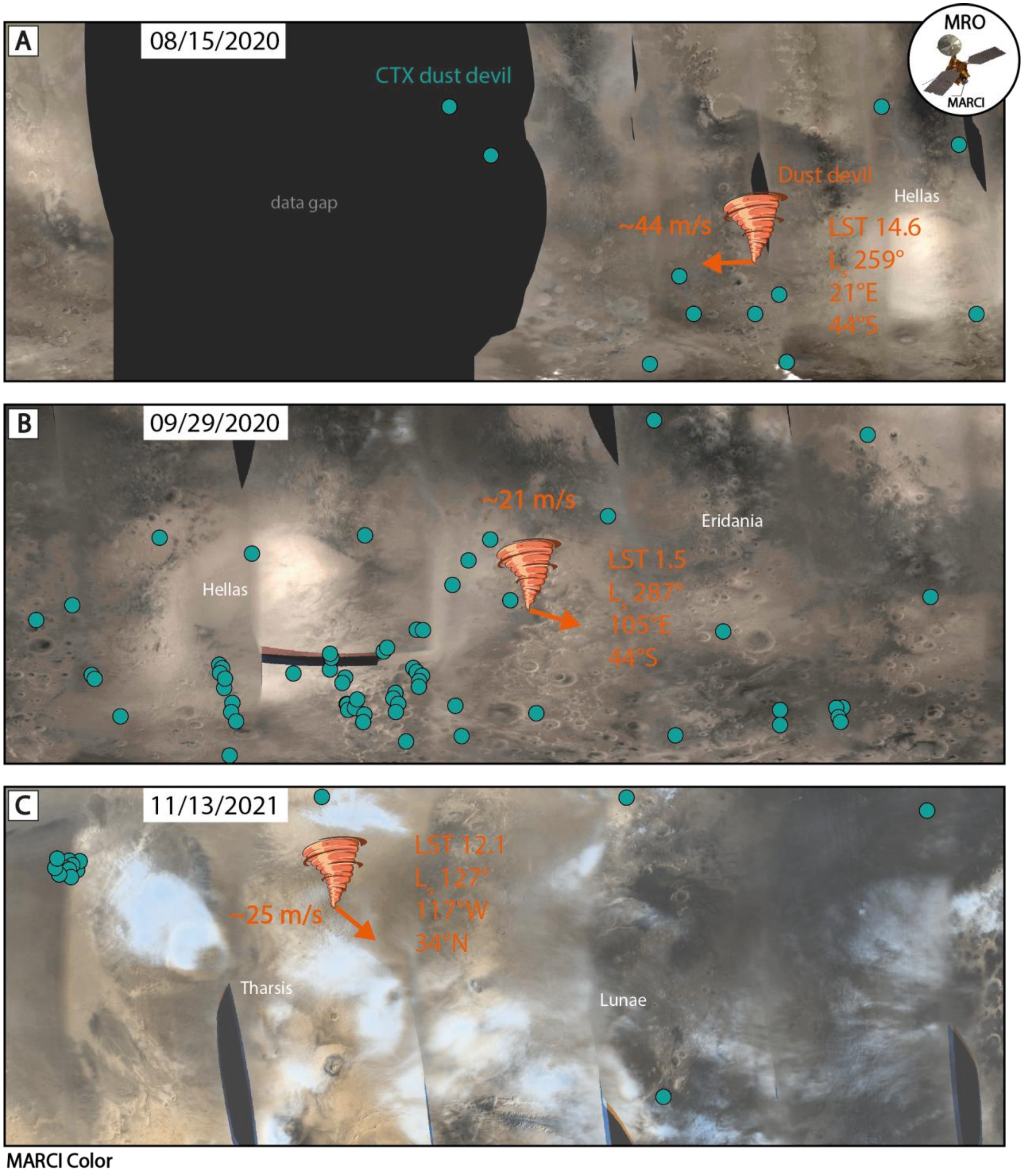

Fig. S12. Fast dust devils without co-located dust storms/clouds or dust lifting events/haze. (**A-C**) Locations (orange shapes, location, LST, L_s, velocity, and azimuth indicated) of fast CaSSIS & HRSC dust devils without a visible co-location to dust storms/clouds or dust lifting events/haze in 2020 and 2021, plotted on MARCI color composites acquired at the same day of occurrence. Note the abundance of large, bright H_2O/CO_2 clouds that are not related to the dust lifting event. Arrow indicates dust devil azimuth (dust devil is located at the tip of the orange vortex). MARCI data gaps are black. Dust devils detected by CTX in the same month (no migration information) are indicated by teal shapes [58]. MARCI image width is ~10,000 km. Image credit: NASA/MSSS/Arizona State University.

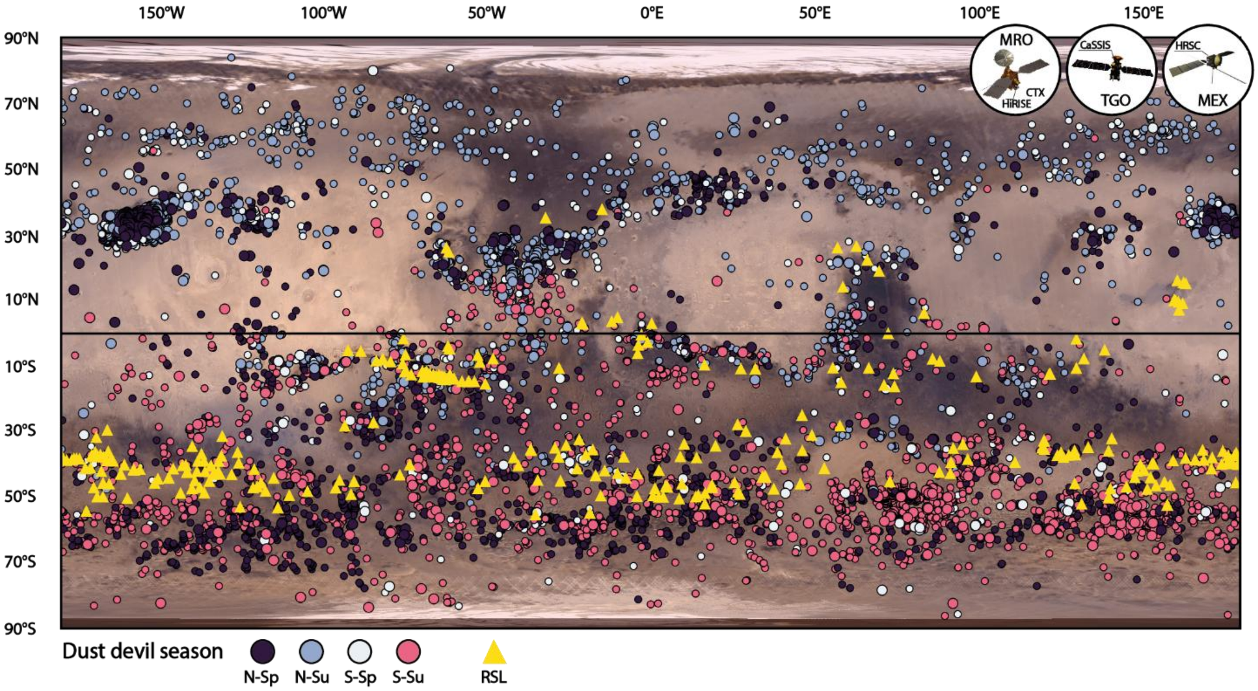

Fig. S13. Spatiotemporal co-location of dust devils and RSL-bearing terrain. Global map of CaSSIS & HRSC dust devil (large shapes, n = 1039) and CTX dust devil (small shapes, n = 12,828 [58]) distribution and season of occurrence. Locations of RSL-bearing terrain indicated by yellow triangles [10]. Viking color mosaic in the background.

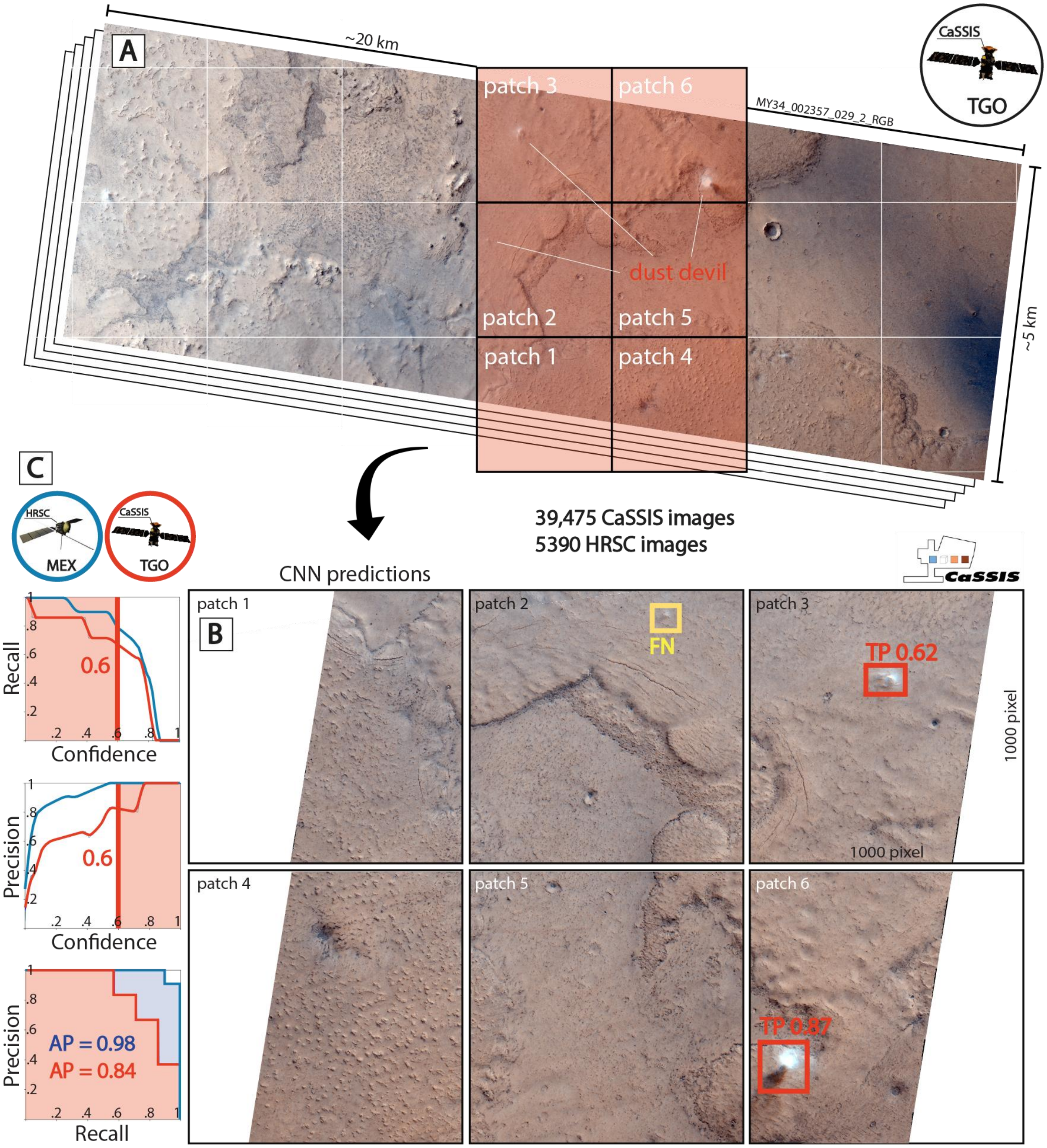

Fig. S14. CNN-driven detection methodology and performance. (**A**) CaSSIS & HRSC images are cropped into 1000x1000 pixel patches and (**B**) ingested into the CNN; correct CNN detections (true positives, TP, red) and missed detections (false negatives, FN, yellow) are indicated; there is no example of a false detection (false positive, FP) in this particular example. (**C**) Performance assessment of the CaSSIS (red) and HRSC (blue) detectors, applied 0.6 score threshold indicated. Image credit: ESA/TGO/CaSSIS CC-BY-SA 3.0 IGO; the shown images were cropped from the original CaSSIS images.

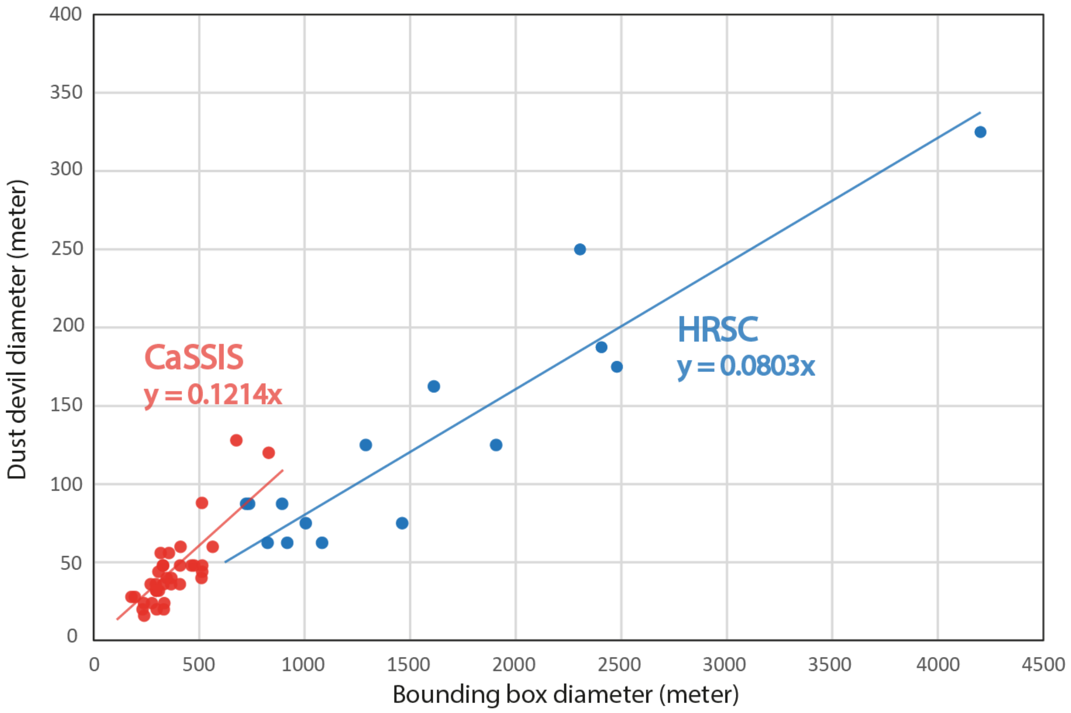

Fig. S15. Calibration of the CNN-derived dust devil size estimates. Correlation of 100 randomly-chosen CaSSIS- (red) and HRSC-derived (blue) dust devil detection bounding box diameters to manually measured diameters (i.e., the physical size of the diameter of the dust devil column where the vortex is liberated as a dust cloud, per *[58]*.

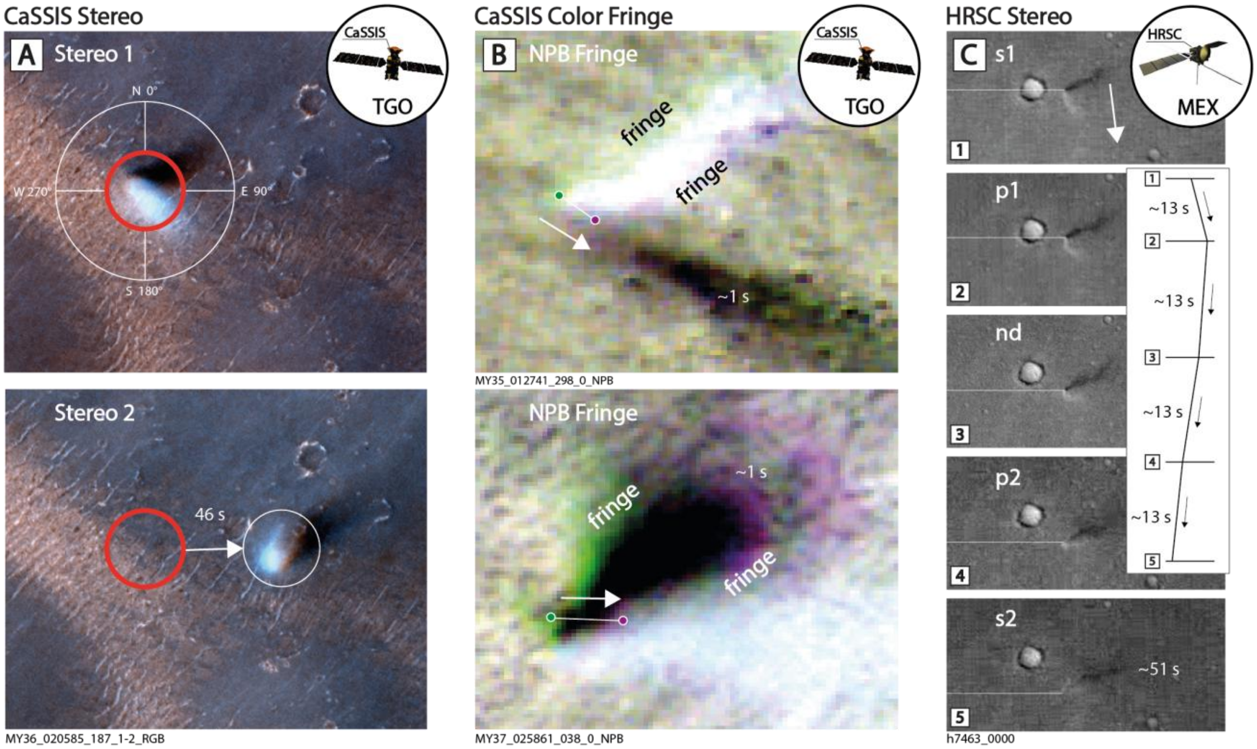

Fig. S16. Dust devil migration measurement techniques. (**A**) CaSSIS stereo observations (n = 2) with a temporal difference of about 46 seconds; (**B**) CaSSIS fringe observations using the NIR and PAN channels (n = 2) with a temporal difference of about 1 second; and (**C**) HRSC stereo observations (n = 5) with a total temporal difference of about 51 seconds. Image credit: ESA/TGO/CaSSIS CC-BY-SA 3.0 IGO, ESA/DLR/FU Berlin CC-BY-SA 3.0 IGO; the shown images were cropped from the original CaSSIS and HRSC images.

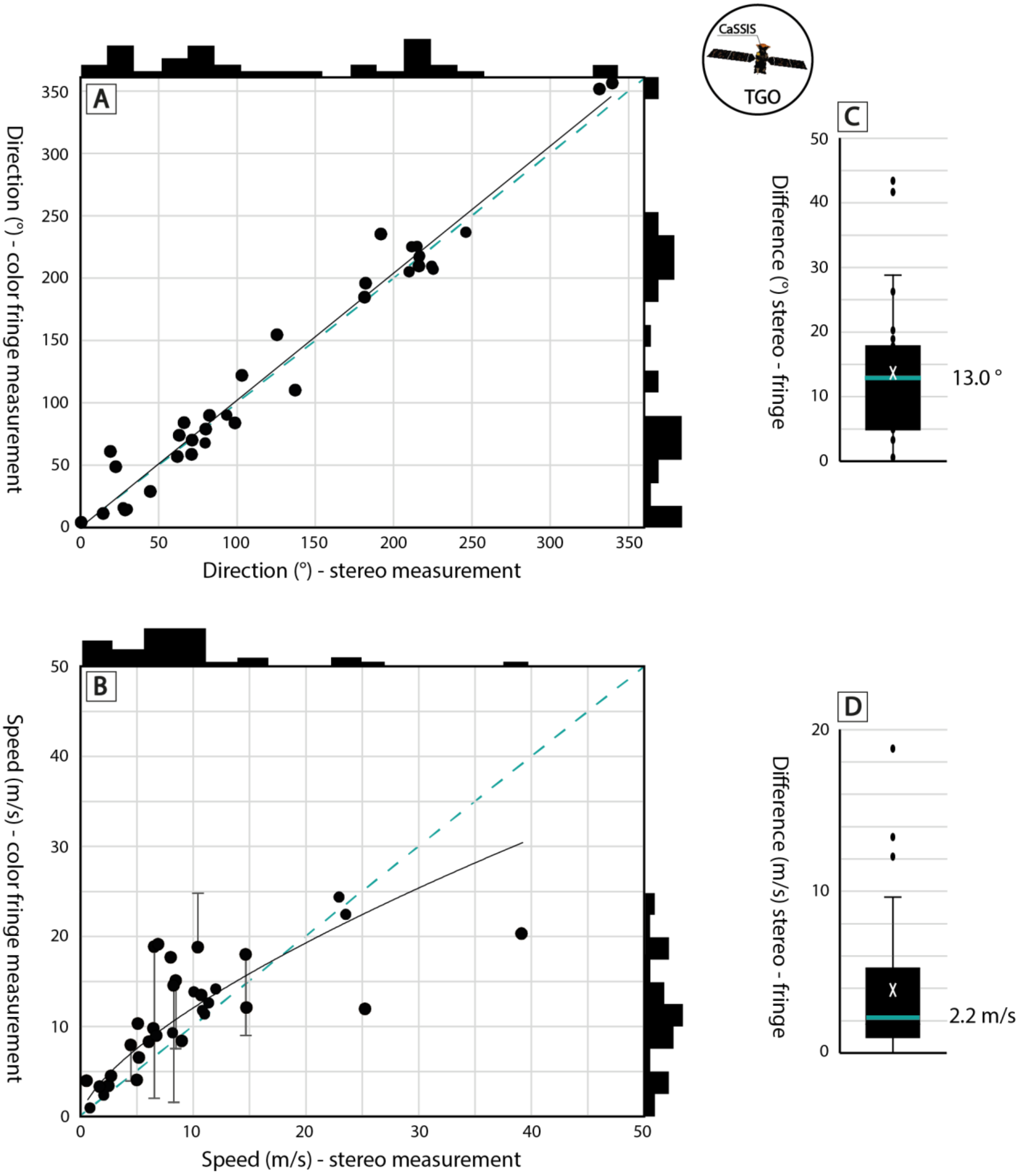

Fig. S17. Cross-comparison of CaSSIS stereo and CaSSIS color fringe migration measurements. (**A-B**) Correlation and cross-comparison of CaSSIS stereo and CaSSIS fringe measurements of velocity and azimuth using 35 dust devils in 14 CaSSIS images; linear/logarithmic fits indicated. (**C-D**) box and whisker plots of CaSSIS stereo – fringe measurements, median indicated.

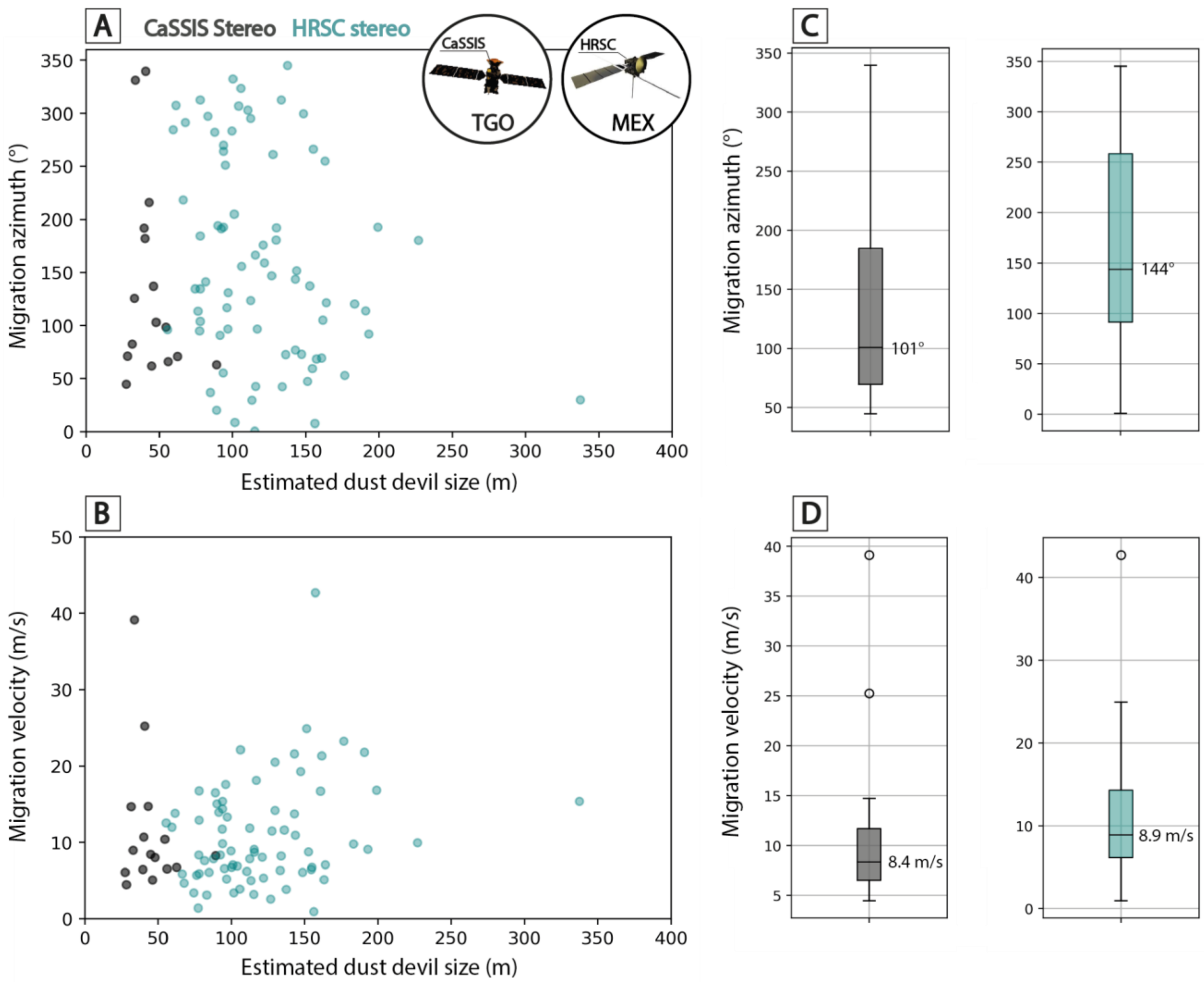

Fig. S18. Cross-comparison of CaSSIS stereo and HRSC stereo migration measurements. (**A-B**) Correlation and cross-comparison of CaSSIS stereo (teal) and HRSC stereo (black) measurements of velocity and azimuth using 91 dust devils. (**C-D**) box and whisker plots of CaSSIS stereo – HRSC stereo differences, median indicated. Note that the azimuth differences are not entirely conclusive as dust devils might move in any direction (both populations represent different dust devils); the values are only reported for completeness.

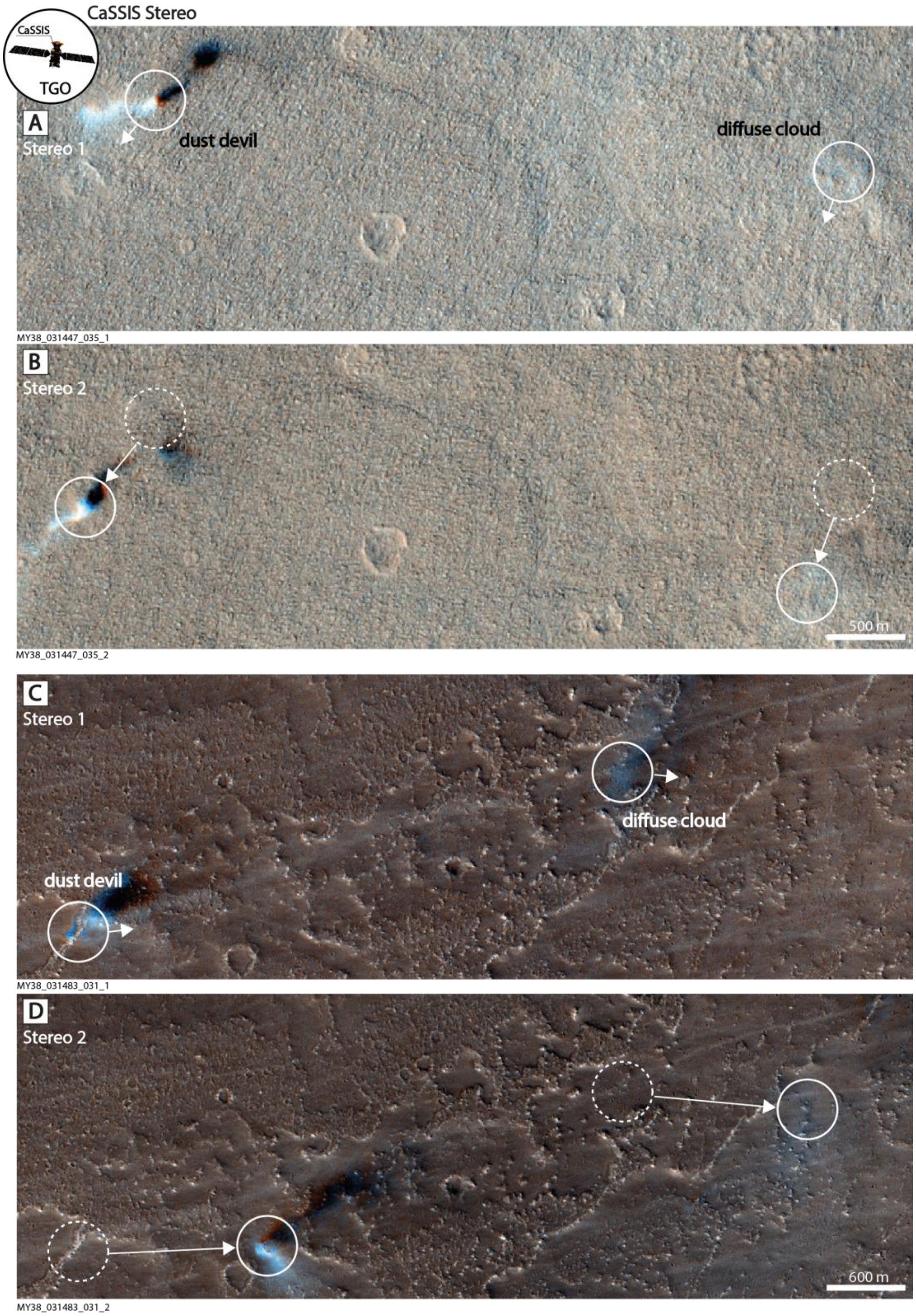

Fig. S19. Observational evidence of dust devil migration with the ambient wind field. (**A-D**) Two examples (stereo 1 – stereo 2, ~49 seconds apart) of dust devils and co-located, diffuse, near-surface clouds (white circles) moving at the same velocity and with the same azimuth (indicated by arrows). Note that the circles might not fully capture the spatial extent of the diffuse clouds. Image credit: ESA/TGO/CaSSIS CC-BY-SA 3.0 IGO; the shown images were cropped from the original CaSSIS images.

Table S1. Summary of all CaSSIS- and HRSC-derived dust devil (DD) measurements as presented in this work.

Property	Value / Range
Number of images scanned	44,865
Number of detected DD	1039
MY scanned	MY 27 to MY 37
Number of DD with velocity & azimuth measurement	373
Max DD velocity	~44 m/s
Mean DD velocity	~18 m/s
DD velocity median StD (HRSC only)	2.3 m/s
DD azimuth median StD (HRSC only)	27°
DD acceleration median StD	0.1 m/s^2
Max wind shear velocity	~2.8 m/s
Max wind stress	~0.1 Pa
Number of DD exceeding wind stress threshold	124 (33 %)
Min DD size	~18 m
Max DD size	~578 m
Mean DD size	~82 m
DD SFD power law exponent	-4.3
Diurnal distribution	8 AM to 5 PM
DD peak occurrence	12:50 to 1:30 PM
Highest DD elevation	6700 m

Table S2. List of all CaSSIS images used for the training and testing of the CaSSIS dust devil detector.
CaSSIS images are openly available here: https://observations.cassis.unibe.ch/

MY35_012439_242_0	MY35_012691_288_0	MY35_012703_293_0	MY35_012713_290_0
MY35_012721_294_0	MY35_012727_287_0	MY35_012728_307_0	MY35_012733_294_0
MY35_012741_298_0	MY35_012751_296_0	MY35_012769_296_0	MY35_010932_208_0
MY36_020677_202_0	MY36_020718_244_0	MY36_020666_200_0	MY36_020639_194_3
MY36_017579_135_0	MY36_017581_143_0	MY36_017606_143_0	MY36_017667_144_0
MY36_017714_145_0	MY36_017717_146_0	MY36_017728_147_0	MY36_017630_146_0
MY34_004659_295_0	MY34_005582_260_0	MY34_005643_250_1	MY34_004836_288_0
MY34_003322_254_0	MY35_011527_295_0	MY34_003338_243_0	MY34_003338_304_0
MY34_003339_306_0	MY34_003342_303_0	MY34_003346_296_0	MY34_003347_299_0
MY34_003416_298_0	MY34_003468_304_0	MY34_004079_246_0	

Table S3. List of all (nadir stereo channel, nd3) HRSC images used for the training and testing of the HRSC dust devil detector. HRSC images are openly available here: https://psa.esa.int/psa/#/pages/search.

h5373_0000.nd3.08	h9528_0000.nd3.07	h9542_0000.nd3.05	hb644_0000.nd3.13
hb651_0000.nd3.07	hb711_0000.nd3.08	hb718_0000.nd3.07	hb725_0000.nd3.07
hd828_0000.nd3.05	hd909_0000.nd3.04	hh707_0000.nd3.04	hh714_0000.nd3.06
hh795_0000.nd3.06	hh802_0000.nd3.06	hh809_0000.nd3.05	hj942_0000.nd3.07
hj949_0000.nd3.06	hj956_0000.nd3.07	hk030_0000.nd3.05	hn578_0000.nd3.02
h0008_0009.nd3.14	h0016_0008.nd3.24	h0018_0000.nd3.24	h0022_0000.nd3.23
h0030_0000.nd3.19	h0038_0000.nd3.19	h0103_0009.nd3.19	h0389_0000.nd3.13

Data S1. The CaSSIS and HRSC dust devil migration dataset. The full dataset of CaSSIS- and HRSC-detected dust devils is available here: https://doi.org/10.48620/87803.

Animation S1. Animation_CaSSIS-fringe.gif. Example of the movement of a dust devil as observed with the CaSSIS color fringe method; 2 frames. Image credit: ESA/TGO/CaSSIS CC-BY-SA 3.0 IGO; the shown images were cropped from the original CaSSIS images.

Animation S2. Animation_CaSSIS-stereo.gif. Example of the movement of a dust devil as observed with the CaSSIS stereo method; 2 frames. Image credit: ESA/TGO/CaSSIS CC-BY-SA 3.0 IGO; the shown images were cropped from the original CaSSIS images.

Animation S3. Animation_HRSC-stereo.gif. Example of the movement of a dust devil as observed with the HRSC stereo method; 5 frames. Image credit: ESA/DLR/FU Berlin CC-BY-SA 3.0 IGO; the shown images were cropped from the original HRSC images.
