# Supplementary figures and images for: Dust devil migration patterns reveal strong near-surface winds across Mars
Source: Sci Adv. 2025 Oct 8;11(41):eadw5170. doi: 10.1126/sciadv.adw5170 (PMC12506970; doi:10.1126/sciadv.adw5170)

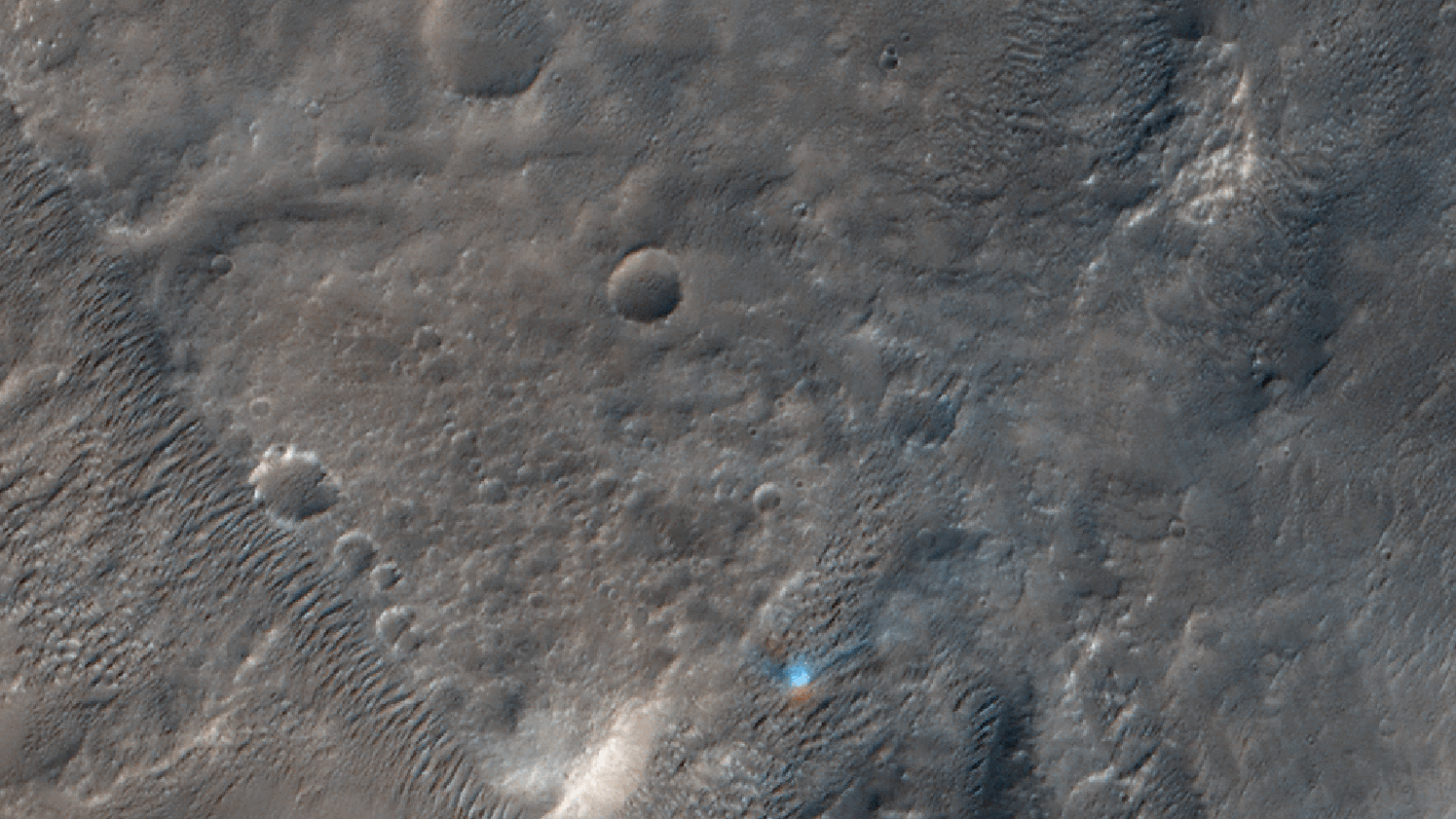

Supplement: Supplementary file 2 — Animations S1 to S3 [file sciadv.adw5170_animations_s1_to_s3.zip › adw5170_animation_s1.gif]

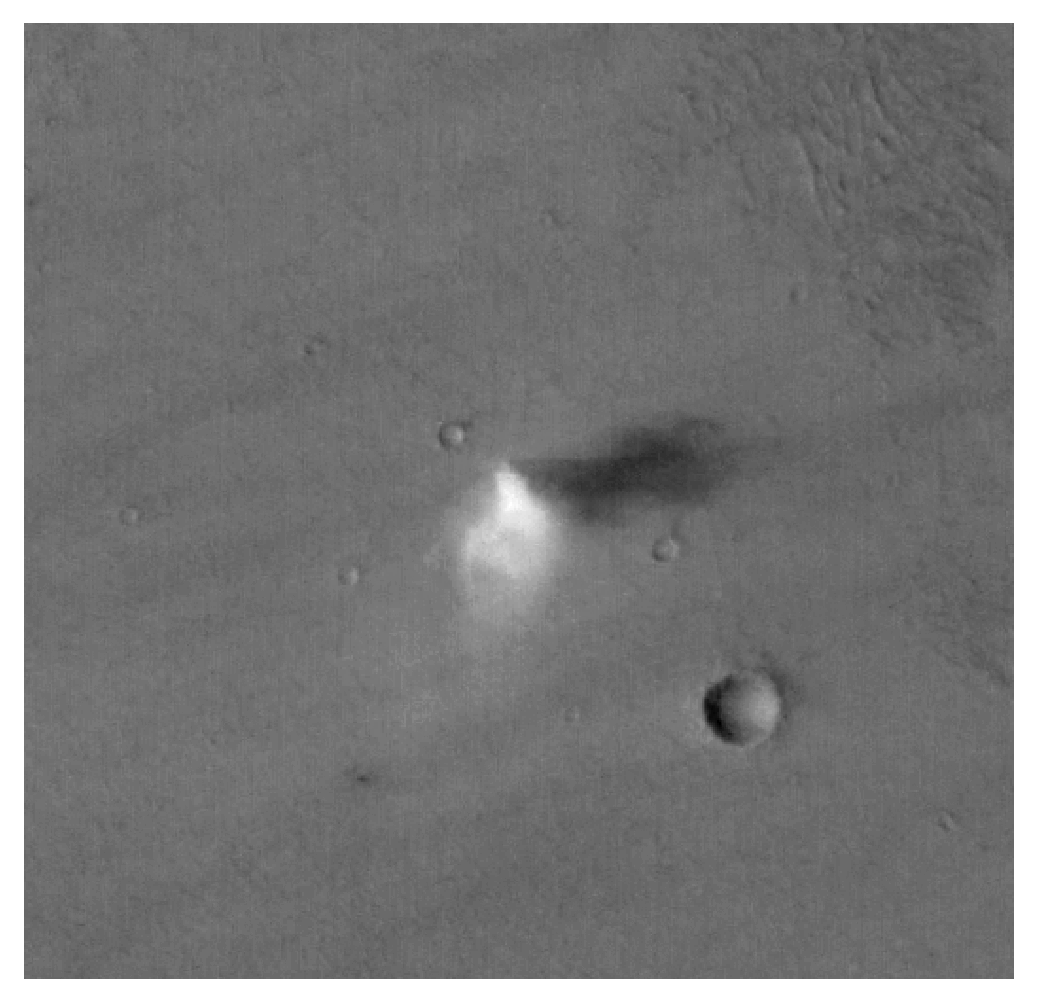

Supplement: Supplementary file 2 — Animations S1 to S3 [file sciadv.adw5170_animations_s1_to_s3.zip › adw5170_animation_s2.gif]

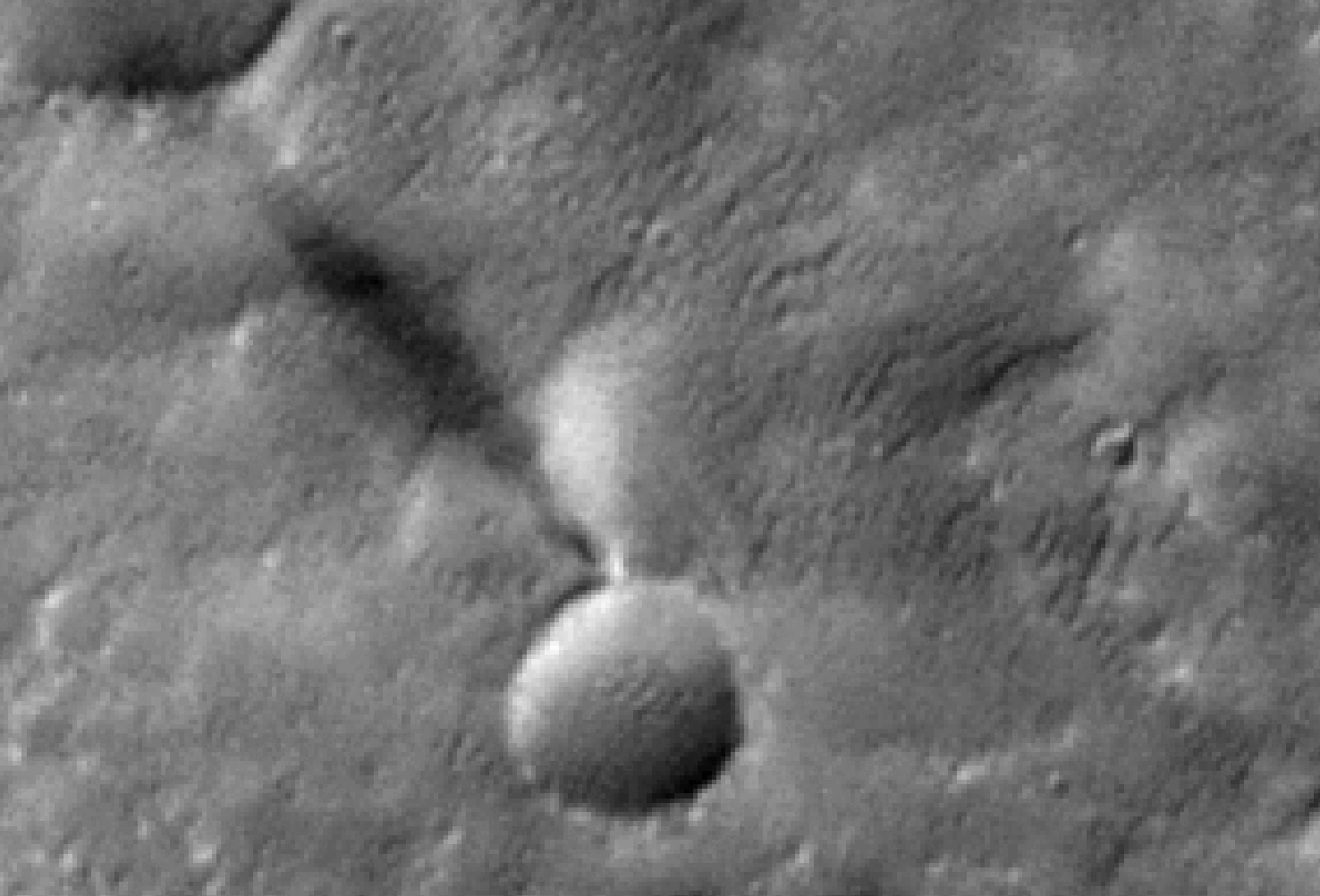

Supplement: Supplementary file 2 — Animations S1 to S3 [file sciadv.adw5170_animations_s1_to_s3.zip › adw5170_animation_s3.gif]
